# Marked Variability in the Extent of Protein Disorder within and between Viral Families

**DOI:** 10.1371/journal.pone.0060724

**Published:** 2013-04-19

**Authors:** Ravindra Pushker, Catherine Mooney, Norman E. Davey, Jean-Marc Jacqué, Denis C. Shields

**Affiliations:** 1 UCD Complex and Adaptive Systems Laboratory, University College Dublin, Belfield, Dublin, Ireland; 2 UCD Conway Institute of Biomolecular and Biomedical Research, University College Dublin, Belfield, Dublin, Ireland; 3 School of Medicine and Medical Science, University College Dublin, Belfield, Dublin, Ireland; 4 EMBL Structural and Computational Biology Unit, Heidelberg, Germany; 5 Centre for Research in Infectious Diseases, University College Dublin, Belfield, Dublin, Ireland; Universität Erlangen-Nürnberg, Germany

## Abstract

Intrinsically disordered regions in eukaryotic proteomes contain key signaling and regulatory modules and mediate interactions with many proteins. Many viral proteomes encode disordered proteins and modulate host factors through the use of short linear motifs (SLiMs) embedded within disordered regions. However, the degree of viral protein disorder across different viruses is not well understood, so we set out to establish the constraints acting on viruses, in terms of their use of disordered protein regions. We surveyed predicted disorder across 2,278 available viral genomes in 41 families, and correlated the extent of disorder with genome size and other factors. Protein disorder varies strikingly between viral families (from 2.9% to 23.1% of residues), and also within families. However, this substantial variation did not follow the established trend among their hosts, with increasing disorder seen across eubacterial, archaebacterial, protists, and multicellular eukaryotes. For example, among large mammalian viruses, poxviruses and herpesviruses showed markedly differing disorder (5.6% and 17.9%, respectively). Viral families with smaller genome sizes have more disorder within each of five main viral types (ssDNA, dsDNA, ssRNA+, dsRNA, retroviruses), except for negative single-stranded RNA viruses, where disorder increased with genome size. However, surveying over all viruses, which compares tiny and enormous viruses over a much bigger range of genome sizes, there is no strong association of genome size with protein disorder. We conclude that there is extensive variation in the disorder content of viral proteomes. While a proportion of this may relate to base composition, to extent of gene overlap, and to genome size within viral types, there remain important additional family and virus-specific effects. Differing disorder strategies are likely to impact on how different viruses modulate host factors, and on how rapidly viruses can evolve novel instances of SLiMs subverting host functions, such as innate and acquired immunity.

## Introduction

The majority of enzymatic (e.g. proteases) and structural (e.g. capsid proteins) viral proteins have defined tertiary structure, but it has emerged over the last number of years that many functions vital for competent viral infection, particularly in terms of host interactions, are mediated by protein regions that lack defined tertiary structure in their native state [1], [2], [3], [4], [5], [6], [7], [8], [9] Their interactions may be mediated by short linear protein motifs (SLiMs) [10] and other recognition domains [11], [12] embedded within the disordered region by longer disordered interfaces referred to as disordered domains [13], or by combinations of motifs and disordered domains [14], [15]. Disordered regions may in some [16], but not all, cases form a secondary structure on binding to their interaction partner. In their utilization of disordered proteins, viruses resemble cellular organisms, in particular eukaryotes, which make extensive use of disordered proteins [17], [18], [19], [20], [21].

There may be particular features of the viral lifestyle that predispose them towards use of disordered regions. Some viruses are encoded within spatially restricted genomes, and being able to map multiple functions to a stretch of protein, facilitated by structural reorganization [22], may be advantageous. The ability to rapidly acquire small motifs made accessible within disordered stretches of protein that manipulate host proteins by interacting with them may also be beneficial under certain circumstances. Since during evolution viruses often cross between quite different hosts, the evolvability^20^ of a virus may be an important feature in certain families. Consequently, disordered regions may enhance the rate of emergence of new phenotypes, in addition to the range of potential phenotypes. The use of compact interfaces allows increased redundancy by allowing functionality to be mediated by a set of short disordered regions (e.g. the Late domains of retroviral Gag proteins) rather than on a single globular interface, resulting in increased evolutionary robustness of viral proteins [10] Thus, a deeper understanding of the role of intrinsically disordered regions in viruses is important if we are to fully understand the relationships between protein sequence and function, and also to understand the relationship between overall proteome organization and long-term evolutionary strategies of different viruses.

There has been some progress to date in elucidating molecular features of disordered regions of viral proteins. Convergently evolved examples for more than 50 of the ∼150 eukaryotic motif types annotated in the ELM database [23] have been experimentally validated in viral proteins [10]. These motifs have roles in manipulating cell signaling, targeting host proteins for proteosomal degradation, directing viral proteins to the correct subcellular localisations, altering transcription of host proteins and deregulating cell cycle checkpoints [10]. For example, the Nef protein of HIV-1 has distinctive motifs mediating interaction with the human proteins NMT1, PCAS-1, Hck, beta-COP, and AP. The Epstein-Barr virus LMP1 protein, has motifs required for binding to TRAF, BTrCP, JAK3 and TRADD proteins, that collectively modulating NF-kB signaling. The Adenovirus E1A protein contains multiple motifs to allow the virus to alter the host gene expression [10].

While the functional importance of intrinsically disordered interactionss in viruses^12^ has been established, to date there is limited understanding of the nature and diversity of such regions across different viruses. SLiMs can be easily and rapidly evolved *de novo*, whereas evolution of a globular interface for viral host protein interaction may not evolve as quickly. Viruses can acquire functional globular domains from their host, however, horizontally transferred genes are typically acquired only by larger viruses that can incorporate a stolen host gene into their genome without having to jettison other proteins that are vital for independent viral function [24]. Therefore, very small viruses may have the most to gain from the multifunctional flexibility and evolutionary fluidity of disordered regions. On the other hand, their genomes may be so constrained to encode structural proteins with largely ordered domains, that may leave little room for accessory proteins with disordered regions to evolve. Intriguingly, some viruses can avoid such restrictions by encoding two or more overlapping proteins in different reading frames. We anticipated that these competing selection constraints would result in different distributions of disordered regions among different viral groups.

For this reason, we set out here to characterize the variation in viral protein disorder across all sequenced viruses, to deepen understanding of disordered regions in determining key features of viral biology. We find a previously under-appreciated striking variation in the extent of disorder among viruses, and we investigate potential reasons why such variability might have arisen.

## Results

To understand disorder in viral proteins, we systematically surveyed predicted protein disorder across many viruses, looking for trends within and between families. We were particularly interested to determine if any of the variation in disorder could be accounted for, in whole or in part, by variation in genome size or by variation in base composition, or by other viral factors such as the type of host they have, or the basic viral type (DNA or RNA, single or double stranded). Since a number of alternative predictors appeared relatively well correlated (Table S4, Fig S13, Results S1), and there was an apparent relationship between predicted and observed disorder for viral proteins (Table S6, Results S1) we relied on IUPRED predictions of disorder for this survey. We also focused on predictions within proteins rather than within precursor polyproteins, noting that the overall survey results were very strongly correlated, regardless which way the analysis was completed (Fig S3).

### Viral genomes have strong variability in intrinsic disorder both between and within viral types and families

**Figure 1:**
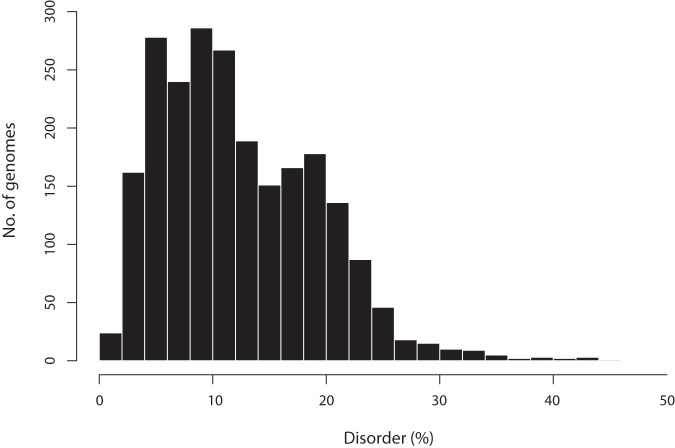
Histogram of predicted intrinsic disorder in 2,278 viral genomes.

**Figure 2:**
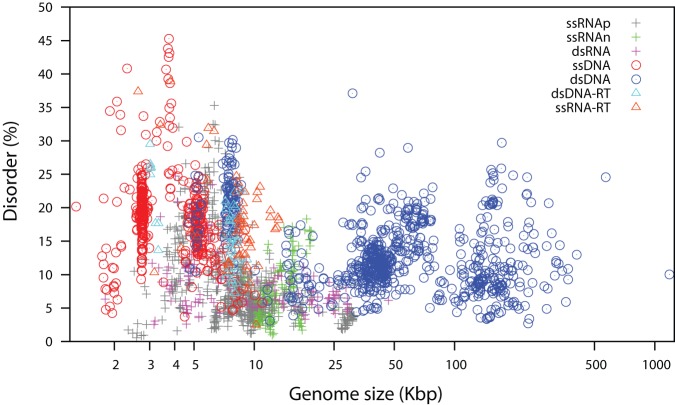
Overall predicted intrinsic disorder in 2,278 viral genomes. The type of genomes shown are: single strand RNA positive strand (ssRNAp), single strand RNA negative strand (ssRNAn), double strand RNA (dsRNA), single strand DNA (ssDNA), double strand DNA (dsDNA), retro-transcribing (double stranded DNA with RNA intermediate, dsDNA-RT; single stranded RNA with DNA intermediate, ssRNA-RT) viruses. “plus”, “circle” and “triangle” are used to represent RNA, DNA and retro-transcribing viruses respectively. The Spearman's correlation coefficient (ρ) between percent disorder and genome size is −0.30 and p<3×10^−16^.

We investigated predicted intrinsic disorder in all viral genomes. The percent disorder (D) for a viral genome represents the percentage of residues which the IUPred software [25] predicts to be disordered, with a cut-off of greater than 0.5, using its short disorder prediction method. The mean percent disorder (µ_D_) for all viral genomes studied was 12.4% with a standard deviation (σ_D_) of 6.9%. We noted that viral genomes have a strikingly high variability in intrinsic disorder, ranging from 0.5–48.5% (Fig. 1 and Fig. 2). Disorder was seen to range extensively between the major viral types, and within them (Table 1; Fig. 2).

**Table 1:** Intrinsic disorder in viral genomes according to genome type.

*Type*	*N* a	*S* b *(kb)*		*D* c *(%)*		*Correlation* d		*R^2^_B_* e	*R^2^_BS_* f	*p-corrected* g
		μ	Σ	μ	σ	ρ	p			
ssRNAp	692	9.96	6.27	8.4	5.9	−0.50	**2.20×10^−16^**	0.46	0.48	**2.82×10^−7^**
dsDNA	803	74.10	80.78	13.3	5.6	−0.27	**1.22×10^−14^**	0.29	0.30	**6.94×10^−5^**
ssDNA	420	4.00	1.62	18.5	6.2	−0.30	**4.95×10^−10^**	0.25	0.34	**5.79×10^−13^**
ssRNAn	133	13.53	2.80	7.4	4.4	0.50	**6.14×10^−10^**	0.25	0.52	**2.77×10^−14^**
dsRNA	129	11.89	8.89	8.7	4.3	−0.33	**1.65×10^−04^**	0.34	0.36	0.03
dsDNA-RT	44	6.96	1.87	16.0	5.5	−0.29	0.05	0.23	0.41	**1.01×10^−3^**
ssRNA-RT	57	8.45	2.39	17.9	7.0	−0.17	0.21	0.24	0.24	0.33
Satellites	112	1.34	0.20	8.5	7.4	−0.45	**7.36×10^−7^**	0.45	0.44	0.73

a Number of genomes in the family.

b Genome size; µ and σ represent the mean and standard deviation respectively.

c Percent protein disorder as defined in Materials & Methods.

d Correlation between disorder and genome size; ρ and p represent coefficient of correlation and probability respectively.

e Proportion of the variance accounted for by base composition.

f Proportion of the variance accounted for by base composition and genome size.

g Corrected probability that genome size is a significant predictor of disorder, from an analysis of variance model including the composition of the four bases as covariates. Significant probabilities after Bonferroni correction are marked in bold.

**Figure 3:**
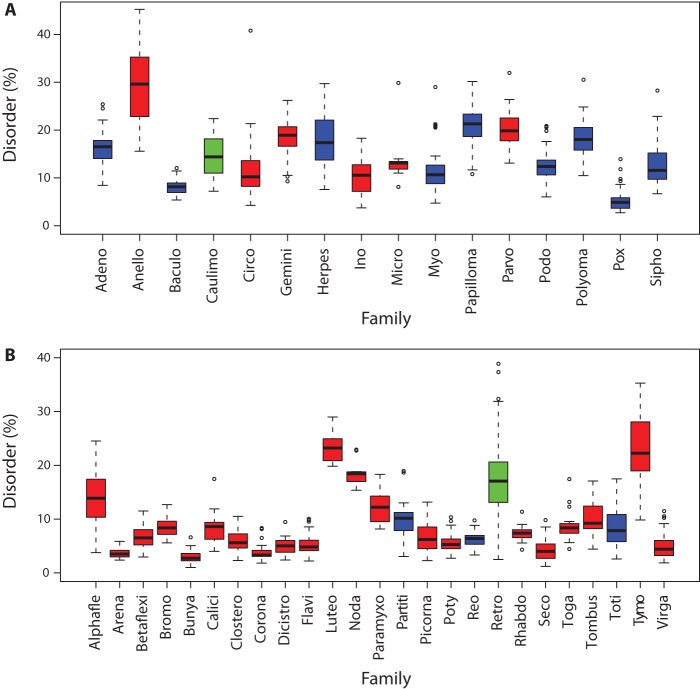
Box plots of predicted disorder for 41 families. Only those families are shown where the number of genomes was greater than 10. **A**) DNA viruses, **B**) RNA viruses. Single stranded viruses are in red, double stranded viruses are in blue and retro-transcribing viruses with DNA or RNA intermediates are in green. Box plots indicate the minimum, maximum, first quartile, third quartile and median for each family's percentage disorder.

Different viral families vary considerably in the extent of sequence similarity and redundancy. Rather than redundancy reduce the dataset, which may serve to reduce the amount of disorder variation in the survey, we instead chose to consider the average disorder shown in each family, and to what extent that showed a similar extensive range in values. A box plot of intrinsic disorder for each family was plotted (Fig. 3), and we tabulated mean intrinsic disorder for all viral families (Table 2). There is a strong between-family variability in intrinsic disorder, with mean intrinsic disorder varying from 2.9% to 29.5%, with a standard deviation of mean family intrinsic disorder of 6.4%. There is considerable variability within families also as summarized by the standard deviation for each family (Table 2; more detailed information on the range and interquartile range for each family is given in Table S5). Some families show more noticeable variation among their members (σ_D_ >5%) namely *Circoviridae*, *Anelloviridae*, *Tymoviridae*, *Retroviridae* and *Herpesvirida*e, while for other families, the extent of protein disorder is very similar across all members.

**Table 2:** Relationship between disorder and genome size within each family.

*Family*	*N* a	*S* b *(Kbp)*		*D* c *(%)*		*Correlation* d		*R^2^_B_*	*R^2^_BS_*	*p* e
		μ	σ	Μ	σ	ρ	P			
*Geminiviridae*	254	3.59	1.20	18.57	3.13	−0.38	**5.0×10^−10^**	0.10	0.22	**2.51×10^−9^**
*Virgaviridae*	38	8.11	2.17	4.93	2.45	0.78	**9.9×10^−9^**	0.13	0.64	**3.70×10^−8^**
*Alphaflexiviridae*	40	6.80	0.81	13.94	4.58	0.57	**1.7×10^−4^**	0.35	0.71	**1.16×10^−7^**
*Podoviridae*	91	42.59	14.18	12.47	2.93	0.29	5.9×10^−3^	0.24	0.35	**2.29×10^−4^**
*Bromoviridae*	29	8.43	0.27	8.52	2.01	0.57	1.5×10^−3^	0.30	0.59	**2.73×10^−4^**
*Bunyaviridae*	25	13.98	2.70	2.90	1.22	0.06	0.77	^−^0.02	0.40	**7.01×10^−4^**
*Myoviridae*	102	109.18	70.18	11.42	3.91	^−^0.29	3.1×10^−3^	0.57	0.61	**1.17×10^−3^**
*Microviridae*	15	5.12	0.65	13.51	4.76	0.05	0.87	0.75	0.87	6.90×10^−3^
*Poxviridae*	27	185.60	51.15	5.63	2.75	0.11	0.57	0.83	0.87	0.01
*Paramyxoviridae*	33	15.86	1.28	12.37	2.97	0.41	0.02	0.14	0.29	0.01
*Potyviridae*	79	9.83	0.41	5.51	1.54	0.06	0.59	0.22	0.27	0.01
*Secoviridae*	32	11.19	1.61	4.16	2.08	0.71	**4.7×10^−6^**	0.37	0.48	0.01
*Coronaviridae*	52	29.30	1.31	3.68	1.33	0.29	0.04	0.32	0.39	0.02
*Picornaviridae*	56	7.67	0.48	6.53	2.51	0.59	**2.2×10^−6^**	0.72	0.74	0.02
*Parvoviridae*	53	5.09	0.55	19.96	3.66	−0.42	1.7×10^−3^	0.30	0.35	0.03
*Tombusviridae*	43	4.27	0.46	10.29	3.12	−0.26	0.10	0.32	0.38	0.04
*Papillomaviridae*	96	7.67	0.33	21.17	4.02	−0.14	0.18	0.46	0.47	0.09
*Closteroviridae*	25	16.31	1.38	5.85	1.98	−0.22	0.29	0.17	0.24	0.10
*Luteoviridae*	22	5.74	0.15	23.08	2.36	0.43	0.04	0.34	0.41	0.11
*Nodaviridae*	12	4.49	0.08	18.51	2.33	0.11	0.74	0.74	0.80	0.11
*Polyomaviridae*	21	5.14	0.17	18.62	4.80	0.40	0.07	0.69	0.71	0.16
*Rhabdoviridae*	27	12.42	1.21	7.32	1.34	−0.08	0.69	−0.01	0.03	0.17
*Adenoviridae*	26	34.85	8.34	16.14	4.59	0.60	1.5×10^−3^	0.74	0.75	0.17
*Partitiviridae*	25	4.25	0.68	9.88	3.74	0.20	0.35	0.32	0.34	0.23
*Siphoviridae*	244	47.79	15.91	12.75	3.91	0.55	**2.2×10^−16^**	0.62	0.62	0.23
*Togaviridae*	17	11.52	0.49	8.78	3.13	0.36	0.15	0.78	0.79	0.26
*Flaviviridae*	52	10.96	1.76	5.23	1.84	−0.15	0.28	0.71	0.71	0.30
*Retroviridae*	57	8.45	2.39	17.94	7.01	−0.17	0.21	0.24	0.24	0.33
*Arenaviridae*	26	10.52	0.15	3.58	0.80	−0.09	0.67	0.12	0.12	0.35
*Reoviridae*	39	23.52	5.87	6.23	1.49	0.16	0.34	0.10	0.09	0.44
*Baculoviridae*	53	131.86	21.97	8.14	1.50	0.47	**4.3×10^−4^**	0.57	0.56	0.48
*Herpesviridae*	42	163.97	43.17	17.87	5.40	0.48	1.4×10^−3^	0.73	0.73	0.49
*Tymoviridae*	21	6.42	0.37	22.83	7.03	0.26	0.25	0.50	0.48	0.53
*Betaflexiviridae*	46	8.28	0.61	6.74	2.06	−0.17	0.26	0.11	0.09	0.63
*Caulimoviridae*	36	7.82	0.44	14.55	4.21	0.08	0.66	0.20	0.17	0.71
*Anelloviridae*	36	3.26	0.54	29.50	8.31	0.26	0.13	0.38	0.36	0.72
*Caliciviridae*	21	7.72	0.54	8.33	2.94	−0.17	0.45	0.15	0.10	0.73
*Dicistroviridae*	14	9.29	0.57	5.17	1.74	0.20	0.48	0.66	0.63	0.76
*Totiviridae*	29	5.67	1.72	8.64	4.31	0.23	0.22	0.39	0.36	0.89
*Circoviridae*	16	2.05	0.41	12.75	8.86	0.07	0.79	0.58	0.53	0.92
*Inoviridae*	26	7.29	1.08	10.43	4.19	−0.09	0.65	0.60	0.58	1.00

a Number of genomes in the family.

b Genome size; µ and σ represent the mean and standard deviation respectively.

c Percent protein disorder as defined in Materials & Methods.

d Correlation between disorder and genome size; ρ and p represent coefficient of correlation and probability respectively.

e Probability that genome size is a significant predictor of disorder, from an an analysis of variance model including the composition of the four bases as covariates. Significant probabilities after Bonferroni correction are marked in bold.

### Weak overall tendency for smaller viral genomes to be more disordered

**Figure 4:**
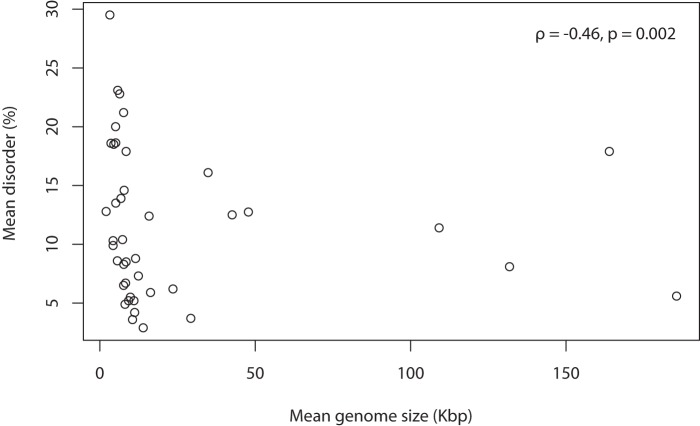
Mean intrinsic disorder versus mean genome size for 41 families represented by more than ten genomes.

We tested whether viral families with smaller genomes tend to have more disordered proteins. The mean genome size varies from 2 kb (*Circoviridae*) to 185kb (*Poxviridae*) (σ_S_  = 43 kb) (Table 2). There is a significant negative correlation between mean intrinsic disorder of each family and mean genome size, considering only an average for each family as a single datapoint in the analysis (Fig. 4). Since distributions of disorder are not normal, we used the non-parametric Spearman's correlation coefficient (ρ) to quantify how the rank of disorder and the rank of genome size are associated (Fig. 4: (; ρ = −0.46, p = 0.002). The extent of this correlation is somewhat reduced when the analysis is instead carried out on all viruses individually (Fig. 2; ρ = −0.30, p<2.2×10^−16^); the latter analysis is weighted in favour of the smaller viruses, which are much more numerous in the dataset. Thus, overall, smaller viral proteomes tend to have more intrinsically disordered residues (Fig. 2). We wished to establish if this effect was strongly independent of other covariates.

### After accounting for base composition, there is no strong overall relationship between disorder and genome size

It has long been recognized that systematic effects on amino acid usage are influenced by base composition [26]. As disordered residues favour G and particularly C rich codons, it is important to tease apart potential confounding of disorder relationships by base compositional effects. In our analyses, we made three inter-related observations: (1) G+C content is correlated with disorder predictions (2) G+C content also relates to experimentally observed disorder and (3) G+C content relates to genome size (see Table S2).

It is of interest to distinguish whether the base composition is higher because of selection on disorder, or *vice versa*, but in general base composition is a strong biasing factor that is seen most strongly at synonymously variable positions, so it is perhaps a simpler explanation that the base composition is driving the differences in protein disorder, rather than the other way around. Table 3 suggests that both effects are present. Firstly, there is a substantial correlation of four-fold degenerate site base composition with predicted protein disorder, in spite of the fact that base composition preferences at these sites do not alter the amino acid sequences. This indicates that base composition is indeed a substantial driver of the extent of predicted protein disorder. The stronger correlations seen for total base composition indicate that it is likely that selection pressures favouring or avoiding protein disorder also have an additional impact on base composition.

**Table 3:** Relationship between disorder and base composition.

	All residues		Four-fold degenerate synonymous residues	
*Base*	*Correlation with disorder*	*p-value*	*Correlation with disorder*	*p-value*
T	−0.48	10^−131^	−0.36	10^−70^
C	0.46	10^−119^	0.37	10^−76^
A	−0.19	10^−20^	−0.14	10^−11^
G	0.15	10^−13^	0.15	10^−13^

Accordingly, we corrected for the effects of base composition in further analyses, to determine if disorder variation had a relationship with genome size that was independent of base compositional effects. Since many viruses have pronounced strand asymmetry which is reflected in unequal frequencies at synonymous positions of complementary bases, we corrected for the frequencies of the four bases, by including them as terms in a multiple linear regression. Thus, to account for the effects of base composition, we then fitted two regression models that accounted for both base composition and genome size. In the first, we regressed disorder on base composition. In the second, we regressed disorder on base composition and on genome size. To assess how much variation in disorder was accounted for by genome size (once base composition is allowed for), we estimated the difference in the variance accounted for by the two models (difference of the adjusted R^2^ values for each model). This difference provides a measure of the importance of genome size in determining the extent of viral protein disorder. We also inspected whether the genome size term from the second model was statistically significant.

The correlation between intrinsic disorder and base composition in all viral genomes (Figure S1) reveals that ‘C’ is the biggest determinant of intrinsic disorder (ρ = 0.40). The regression model that accounted for both base composition and genome size in all viruses revealed that 17% of variance in disorder was explained by both base composition and genome size, and that genome size remained a significant predictor of disorder (p = 1.6×10^−3^). When we repeated this analysis on family means (that is, we regressed mean disorder of each family on mean genome size, including mean base compositions as a set of four additional covariates), we found that 30% of the variance in disorder is determined by base composition and genome size in families, but that genome size was not a significant predictor of disorder (p = 0.90). Thus, the overall trend noted initially of smaller viral families having more disorder is closely allied with the effects of base composition on genome size.

### Within major viral types, disorder relates strongly with genome size

**Figure 5:**
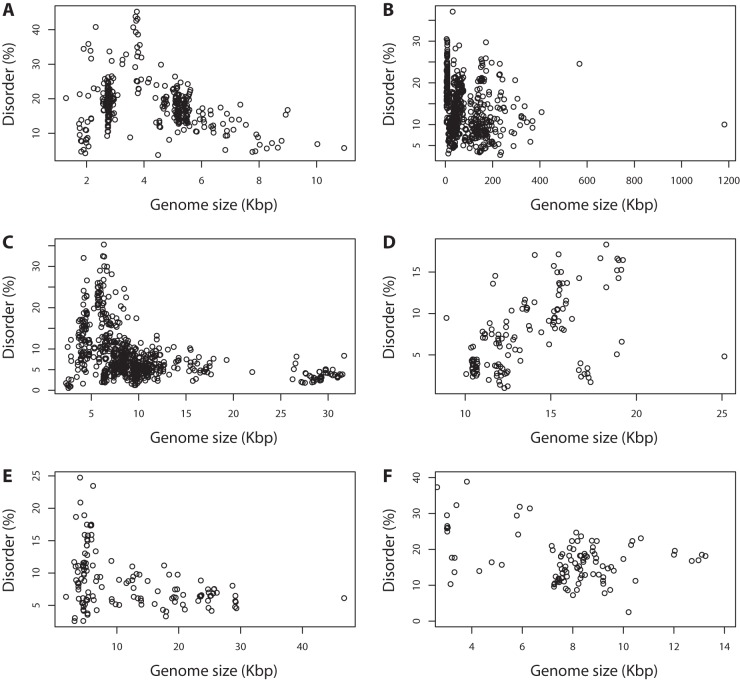
Intrinsic disorder in different types of viral genomes. A) Single stranded DNA, B) Double stranded DNA (dsDNA), C) Single stranded RNA, positive strand, D) Single stranded RNA (ssRNA), negative strand, E) Double stranded RNA and F) Retro-transcribing viruses (dsDNA with RNA intermediate or ssRNA with DNA intermediate).

We investigated whether the level of intrinsic disorder was related to genome size within the major viral types. For these analyses, we concentrated on the findings once base composition was allowed for in the model (Table 1). Mean intrinsic disorder varied from 7.4% (ssRNAn [9]viruses) to 18.5% (ssDNA viruses). Mean genome size for different genome types ranged between 4 kb (ssDNA) and 74kb (dsDNA). Single stranded DNA (ssDNA), double stranded DNA (dsDNA), single stranded RNA positive strand (ssRNAp) and double stranded RNA (dsRNA) viruses show higher intrinsic disorder when the genome size is smaller (Table 1; Fig. 5). Single stranded RNA negative strand (ssRNAn) viruses have genome sizes ranging between 2 kb and 26 kb, and intrinsic disorder significantly increases with the genome size (ρ = 0.50, p = 6.1×10^−10^; Fig S4, Fig. S5).

### Within viral families, both positive and negative correlations are seen with genome size

**Figure 6:**
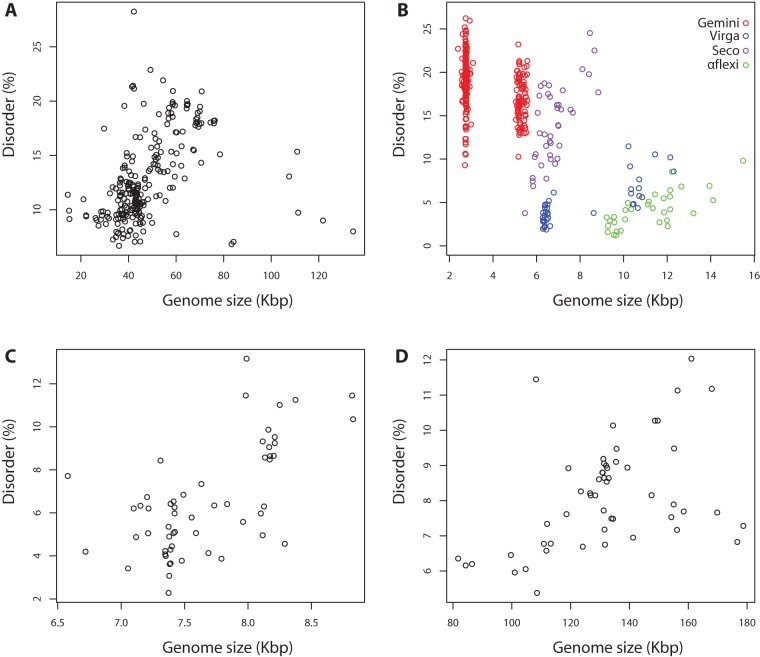
Intrinsic disorder at family level in viral genomes. Families shown are **A**) *Siphoviridae*, **B**) Plant viruses from *Geminiviridae* (Gemini), *Virgaviridae* (Virga), *Secoviridae* (Seco), plant and fungi viruses from *Alphaflexiviridae* (Aflexi) **C**) *Picornaviridae* and **D**) *Baculoviridae*. Except for *Geminiviridae*, all families shown here show a positive correlation of disorder with genome size.

We investigated the relationships between viral disorder and genome size within each viral family (Table 2). While some of the families are relatively small, or have little variability associated with relatively recent common genetic origins, for others there is a large enough variation to permit some interpretation. In a number of families, genome size was a significant determinant of disorder, and accounted for an appreciable proportion of the variation in disorder even after correcting for base composition. These were the *Geminiviridae*, *Virgaviridae*, *Alphaflexiviridae*, *Podoviridae*, *Bromoviridae*, *Bunyaviridae* and *Myoviridae*. Out of these families, *Geminiviridae* and *Myoviridae* families had negative correlations between disorder and genome size, whereas the others had positive correlations. Figure 6 illustrates these trends for a selected set of families.

### Relationship between host and viral disorder

Given an apparent substantial variation of disorder among viruses with different hosts (Table S1, Fig. S2), we wished to determine if there was any correlation of viral and host disorder. Fig S12 indicates no such correlation. We were also interested whether the range of disorder in viruses is greater than that seen in their hosts. Table 4 suggests that this is indeed the case for multi-cellular organisms (invertebrates, plants and vertebrates) which showed a range of viral disorder that was typically double that of their hosts. While bacterial virus disorder spanned a similar range to that of the bacterial hosts, it was curious to note that fungal viruses show a similar span, but a tendency towards lower disorder, so that the range for fungal viruses is 0.5% to 23%, while their hosts lie in the range of 9% to 32%. The most striking range is seen for vertebrate viruses, from 1.8% to 45.2% disordered, in spite of the relatively limited range of vertebrate host disorder. It is clear that viruses are in many ways independent of their hosts in terms of their propensity for protein disorder.

**Table 4:** Comparison of disorder in viruses and hosts.

	*HOST DISORDER* a				*VIRAL DISORDER*				
*Host*	*Mean*	*Min*	*Max*	*Range*	*N*	*Mean*	*Min*	*Max*	*Range*
Bacteria	7.4	2.3	19.5	17.2	108	11.9	3.2	20.9	17.7
Fungi	21.5	9.0	32.5	23.6	19	6.3	0.5	22.7	22.2
Fungi/Protozoa	19.7 b				29	8.6	2.6	17.5	14.9
Invertebrates c	17.6	11.2	23.7	12.5	90	8.6	2.4	28.9	26.5
Plants	20.1	11.5	28.9	17.4	638	12.9	1.2	35.3	34.1
Plants/Fungi	20.8 b				71	11.9	3.0	24.5	21.5
Vertebrates d	21.1	17.6	30.3	12.7	590	13.6	1.8	45.3	43.5
Vert/Invert	19.6 b				108	15.3	2.7	32.0	29.3
Vert/Invert/Plants	19.6 b				66	6.7	3.3	11.4	8.1
Vert/Plants	20.6 b				25	2.9	1.0	6.6	5.6

a From Schad et al. [46].

b mean of other groups presented elsewhere in the table.

c Protostomia.

d Deuterostomia.

### Towards an integrated model explaining disorder variation in viral proteomes

Clearly, there are a number of determinants of disorder in viruses: genome size, base composition, viral type effects, host effects and family-specific effects. Can we therefore say overall, how important these different factors are? One approach is to integrate all these terms into a single statistical model, and investigate the proportion of variance assigned to each factor. Accordingly, we carried out an analysis of variance which incorporates these various factors.

Quantitative predictor variables in the model were base composition (A, T, C and G) and genome size (S) whereas categorical predictors were genome type (Type), host (H) and family (F). In this unified model, the proportion of variance accounted for by the base composition parameters alone was 16.6%; a list of all predictors of disorder and corresponding R^2^ values are shown in Table S3. Family seems to be the single strongest predictor of disorder (74%). Thus, it appears that the most important determinant of how much disorder a virus will have is the family to which it belongs. While a substantial component of this may be down to what broad viral type the family belongs to (36%), clearly there are additional factors. Genome size modeled as a single variable across all viruses adds comparatively little to this model, nor does it add much when other covariates are included (Table S3). This is not too surprising, given that the correlations within some families are positive but within other families are negative (Table 2), so that they will cancel out within the overall model. Overall, over 80% of disorder variation can be accounted for by family and base composition. Since we know that genome size is correlated both negatively and positively with disorder within certain families, it is likely that some of the remaining 17% of variance can be accounted for by family-specific genome size effects, as well as other factors. Thus, between family variability is striking, but there remains substantial within family variability. It appears from these models that individual viral families have distinctive degrees of disorder, most likely reflecting both adaptive and mutational factors. The challenge is to identify these factors, and gain a better understanding of constraints on viral protein function.

We also inspected whether disordered proteins were enriched in viruses with more overlapping reading frames. While smaller genomes with greater disorder have more overlapping genes in ssRNAp and ssDNA viruses (Results S1; Fig S4, S5, S6, S7, S8, S9) the greater disorder among larger genomes of ssRNAn viruses is unlikely to reflect differences in the extent of overlapping genes (Results S1; Fig S10, S11).

## Discussion

We have surveyed 2,278 viral genomes to investigate how intrinsic protein disorder varies at different levels. This analysis has unveiled a very substantial variation among viruses in their predicted disorder. Previous studies [2], [4] had highlighted the fact that many viral proteins are highly disordered, (with most disorder among RNA-binding proteins, moderate disorder among DNA binding proteins, and order among enzymes and multipass membrane proteins [1]). One broad survey of disorder in viruses with a similar interest to our study [9] gave some consideration to this range of variation in viral disorder. They highlighted that certain proteins have high levels of disorder, pointing out that more than 20 small viruses have over 50% disorder, and that for larger viruses disorder ranges typically between 20 % and 40%. However, they also noted that certain viruses have very low disorder, such as the human coronavirus NL63 (estimated 7.3% disordered residues [9]). One of the biggest challenges facing our understanding of the dynamics of disorder in viral proteomes is to understand why there are some viruses which strongly avoid disorder.

It is well known that there is substantial variation among the main kingdoms of life in their distribution of disorder. Intrinsically disordered regions are more common among eukaryotic genomes than prokaryotic genomes [27], [28], with putative long (>30 residue) disordered segments found to occur in 2.0% of archaean, 4.2% of eubacterial and around a third of eukaryotic proteins [28], [29], [30]. We might, therefore, have expected that viruses would follow their hosts, a trend discussed in a previous survey of viruses [9]. However, from our analysis, the marked viral variation does not clearly or even substantially follow these main phylogenetic trends of their hosts. Thus, the large double stranded poxviruses and herpesviruses showed markedly differing levels of predicted disorder, in spite of the similarity of their hosts. The predicted disorder among viruses of different hosts showed no clear pattern, with prokaryotes having both high disorder (eubacteria, 17.9%) and low disorder (archaea, 6.3%) groups, and with similar variation among the viruses of various eukaryotic hosts (Table 4). Clearly, a simplistic correlation of disorder with “complexity” does not fit well with any measures of either viral or host complexity, and it is likely that the distribution of disorder is influenced by multiple factors. Such factors may well include aspects of latency, virulence, life cycle and selection pressures among mutated viral copies within a cell [31].

Overall, smaller genomes tend to have more disordered residues, but this weak trend is largely absent when base composition is taken into account. We hypothesized prior to commencing this study that this would be the case, on the grounds that disordered extended proteins have a larger interaction surface with which they may come in contact with, and modify the function of, host proteins [32]. However, this effect was rather small over all viruses, and was largely removed when correcting for base composition as a potential explanatory factor. When we looked within viral families, we found much more striking correlations between genome size and protein disorder. In a number of families, there was a clear negative correlation between disorder and genome size (even after allowing for base composition), consistent with our original hypothesis, but in a few families there was a positive correlation. How can we account for this? We conclude that there are family-specific determinants of disorder, that relate in some families in part to genome size, in ways that have yet to be identified. Such factors could include mutation rates, pathogenicity and preference for transient versus persistent infection, which may alter the requirement for accessory host interaction proteins. Relationships between genome size and disorder among some bacteria may be consistent with simplification for smaller genomes and less disordered proteins [33], although it is difficult to determine causation. However, without a much fuller knowledge regarding the adaptive and non-adaptive constraints on a wide range of different viruses, it is difficult to identify the precise components of different viral evolutionary lifestyles that are most likely to have given rise to such strikingly different degrees of protein disorder.

Increases or decreases in disorder in viral protein evolution may arise through a number of mechanisms, ranging from addition and deletion of largely disordered proteins, to insertion and deletion of short or medium length disordered regions, to gradual shifts between more ordered and disordered states of proteins; and including the introduction of novel proteins encoded in alternative reading frames [34]. Inspection of the most disordered proteins (Table S7) reveals that they have roles not only in cellular signaling, such as the HIV1 *Nef* protein, but also in viral structure and movement. The apparent relationship between disorder and base composition suggests that the pattern of mutation, which is the likely main driver of base composition, itself has an influence on the extent of protein disorder. It is unclear to what extent the mutation rate itself may be a partial driver of the extent of disorder [35]: while pox viruses appear to have more rapid mutation rates and lower disorder compared to herpes viruses [36], these two observations may or may not be linked by direct or indirect causation. A gold standard set of mutation rates for different viruses would help to address this important question, but no such dataset of viral mutation rates exists, and mutation rates cannot be inferred from sequence data as there is no global outgroup alignable to viral proteins shared across many families.

Viral “mimicry” of protein functions often involves direct hijacking of the gene encoding a host protein [24]. For viruses which move easily amongst different hosts, there may be much greater opportunities for such hijacking of functions. One might anticipate that viruses with more disordered proteins may rely more on mimicry of short motifs, rather than hijacking of large protein domains. Amongst the large Pox and Herpes viruses, it is noticeable that although there may be a similar number of documented instances of gene hijacking amongst both groups [37], the much more disordered Herpesviruses have noticeably more documented instances of linear motif mimicry [10]. Thus, the extent of disorder is likely to alter the strategies employed by different viruses in subverting host functions.

The potential deleterious effects of disordered proteins on the efficient functioning of a cell may well be a partial determinant of the particular strategy adopted by a virus, in terms of the selective advantages versus disadvantages of disordered proteins. On the one hand, disordered regions have the potential to increase the physical surface area for interaction with host factors, providing greater subtlety of signaling and rapidity of evolution of novel work-arounds that evade evolving host defences. On the other hand, the disordered proteins themselves may possibly tend to disrupt functioning of both the host cell and perhaps even aspects of virus particle assembly, given the observed deleterious effects of over-expressed disordered proteins [38], [39]; thus, there may be a complex balance between the costs and benefits of any given disordered region.

This survey is wide-ranging, and is likely to be subject to certain biases arising from the historical processes that defined not only viral evolution, but also the science of virology, where the classification of viral families has been based on a wide range of criteria. More detailed studies will be required to fully ascertain the impact of disorder on function, most likely carried out within particular families, complementing computational predictions of disorder with experimental investigations.

One key goal of understanding disordered sequences and their embedded motifs is the targeting of those motifs in order to modulate protein–protein interactions by small molecules [40], [41] or therapeutic peptides. Short motif interactions have the virtue from the viral perspective of being easily evolved *de novo* by mutation of a small stretch of disordered protein, to enable interaction with a new host protein. Such critical interaction points within disordered sequences may play a role both in viral structure and in virus-host interaction (Table 4). Our study provides a clear framework in which to understand the background distribution of disorder in different viruses. This will provide important insights in targeting the most interesting disordered regions. For example, regions of disorder that occur in viruses which tend in general to avoid disorder may be of particular interest, since such disordered regions may play key roles in viral function.

## Materials and Methods

### Sequence Collection

Protein sequences for all viral genomes were retrieved on 10^th^ May 2010 from NCBI Entrez database (http://www.ncbi.nlm.nih.gov/Entrez/). A total of 78,317 protein sequences were obtained from 2390 viral genomes. The Baltimore classification [42] of viruses that classifies viruses by their genome types and replication strategies was used to divide viruses in seven classes. There were 420 single strand DNA (ssDNA) viruses, 803 double strand DNA (dsDNA) viruses, 692 single strand RNA positive strand (ssRNAp) viruses, 133 single strand RNA negative strand (ssRNAn) viruses, double strand RNA (dsRNA), 57 ssRNAp with DNA intermediate (ssRNA-RT) and 44 dsDNA with RNA intermediate (dsDNA-RT). There were 112 satellite viruses which were not used for the study; thus reducing the number of genomes to 2278. Taxonomy for all viruses and host association were retrieved from ICTVdB – The Universal Virus Database available at http://www.ictvonline.org and cross-checked with ViralZone [43], a manually reviewed virus–host web portal available at http://www.expasy.org/viralzone/.

We plotted the genome size versus the number of residues (Fig. S14) to help identify the extent to which statistics may be biased by poorly annotated genomes. These outliers are from the Phycodnaviridae (Chlorella viruses), Nimaviridae (Shrimp White Spot Syndrome Virus), Polydnaviridae (Bracoviruses and Ichnoviruses). For a group of Chlorella viruses, the encoded residues exceeds expectation (Fig. S14). It is noted that many shorter ORFs are likely over-predictions of non-translated putative ORFs [44] and so any investigations of predicted disorder within this family would need to be interpreted with some caution, given that five of the ten members of the Phycodnaviridae showed this excess. Only one other virus showed a marked excess of amino acids predicted, the Shrimp white spot syndrome virus, which is the only representative of the Nimaviridae. For all five members of the Polydnaviridae, there was a deficit of open reading frames relative to expectation. All three of these families were excluded from further analyses, as we made the decision to exclude all families that had ten or fewer members.

### Intrinsically disordered proteins

To predict intrinsically disordered regions of proteins, IUPred [45] was used which predicts intrinsic disorder by estimating the total pair-wise interaction energy, based on a quadratic form in the amino acid composition of the protein. IUPred provides a position-specific score that characterizes the tendency of a given amino acid to fall into an intrinsically ordered or disordered region. For each protein, the IUPred score was generated for each residue in that protein. Each residue was defined as ordered or disordered depending on the IUPred score (S) to be either less than IUPred score threshold (S_Th_  = 0.5) or more respectively. This procedure was repeated for all proteins in a viral genome. Percent intrinsic disorder (D_i_) for i^th^ protein is defined as the ratio of the number of residues with IUPred score above S_Th_ and the length of that protein (L_i_) given by Equation 1.
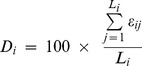
(1)where



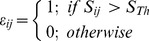
(2)Similarly, D for the whole genome with N proteins is calculated as the ratio of the number of residues having IUPred score above S_Th_ and the total number of residues in the whole genome (Equation 3).
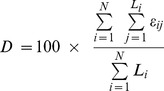
(3)


### Polypeptides

Cleaved polypeptides corresponding to each polyprotein were derived from NCBI GenBank files. For each polyprotein, disorder was calculated in two ways, first by calculating disorder for the whole polyprotein and secondly by calculating the number of disordered residues for all corresponding cleaved polypeptides and dividing by the total length of cleaved polypeptides. Disorder values from the first method were used unless otherwise specified.

### Statistical analyses

Basic statistical analyses were carried out using the R statistical package (http://www.r-project.org). SPSS 15.0 for Windows (Release 15.0.0 2006) statistical software (Chicago: SPSS Inc.) was used for Analysis of Variance (ANOVA) studies. Stata software (StataCorp. 2009. *Stata Statistical Software: Release 11*. College Station, TX: StataCorp LP.) was used to calculate residuals and to create residual plots. Spearman's non-parametric correlation coefficient ρ statistic was used to estimate a rank-based measurement of correlation between genome size and percent disorder. The probability that ρ departed significantly from the expectation of zero for each association was calculated and reported. To test whether intrinsic disorder is predicted by base composition or genome size, multiple regression analysis that uses an Ordinary Least Square (OLS) method was performed.

To see how much variance in disorder is explained by different factors, an Analysis of Variance (ANOVA) was performed to compare the means of each predictors using Univariate General Linear Model (GLM) in SPSS. Disorder was treated as the dependent variable and categorical variables such as type, family, host etc. along with continuous variables of base composition and genome size were fitted as independent variables in the model. The adjusted R^2^ values from each model were tabulated for comparison.

## Supporting Information

Figure S1 **Correlation between intrinsic disorder and base composition.** Bases are **A**) Adenine, **B**) Thymine, **C**) Guanine and **D**) Cytosine.
(EPS)

Figure S2 **Intrinsic disorder in viral genomes based on their host of infection.** Different hosts of infections shown are **A**) Plants, **B**) Vertebrates, **C**) Vertebrates/Invertebrates, **D**) Invertebrates, **E**) Bacteria and **F**) Bacteria and Archaea.
(EPS)

Figure S3 **Disorder in polyproteins.** **A**) Percent disorder for each polyprotein compared to corresponding cleaved polypeptides. **B**) Percent disorder for the whole genome calculated by considering polyproteins and cleaved polypeptides.
(EPS)

Figure S4 **ssRNAp plot of residual (disorder, once composition of the four bases has been corrected for) versus genome size. Labels represent truncated family names.**
(EPS)

Figure S5 **Contrasting genome organisation of different ssRNAp viruses.** The images reflect the representatives of (A) the *Tymoviridae* (Turnip yellow mosaic virus), (B) the *Alphaflexiviridae* (Potato virus X) and (C) the *Luteoviridae* (Luteovirus), contrasted with (D) the larger genome of a *Coronaviridae* representative (MHV). Images are adapted with permission from www.expasy.ch/viralzone.
(EPS)

Figure S6 **ssDNA plot of residual (disorder, once composition of the four bases has been corrected for) versus genome size. Labels represent family names.**
(EPS)

Figure S7 **Contrasting genome organisation of different ssDNA viruses.** (A) An ssDNA virus that is short and disordered, with gene overlaps (*Annelloviridae*). (B) An ssDNA virus that is longer and more ordered, with no gene overlaps (*Inoviridae* represented by M13). (C) The multi-component genome of a larger and more ordered virus, with no gene overlaps (*Nanoviridae* represented by FBNYV). Images are adapted with permission from www.expasy.ch/viralzone.
(EPS)

Figure S8 (a) dsDNA plot of residual (disorder, once composition of the four bases has been corrected for) versus genome size. Labels represent truncated family names.
(EPS)

Figure S9 **dsDNA-RT plot of residual (disorder, once composition of the four bases has been corrected for) versus genome size. Labels represent truncated family names.**
(EPS)

Figure S10 **ssRNAn plot of residual (disorder, once composition of the four bases has been corrected for) versus genome size. Labels represent truncated family names.**
(EPS)

Figure S11 **Contrasting genome organisation of different ssRNAn viruses.** (a) ssRNAn: larger genome, high disorder, some overlap (*Paramyxoviridae*), (b) ssRNAn: smaller genome, less disorder, no overlap (Arenaviridae), (c) ssRNAn: smaller genome, less disorder, some overlap (*Bunyaviridae*). Images are adapted with permission from www.expasy.ch/viralzone.
(EPS)

Figure S12 **Relationship between viral and host disorder (see Table S4).**
(PDF)

Figure S13 **Comparison of different methods of disorder prediction for each virus in the dataset.**
(PDF)

Figure S14 **Predicted number of amino acid residues versus genome size, to highlight potential annotation errors in the dataset.**
(PDF)

Table S1 Intrinsic disorder in viral genomes classified by the hosts they interact with.
(PDF)

Table S2 Variance in genome size explained by base composition.
(PDF)

Table S3 Percentage of variance in disorder (Adjusted *R^2^* for the rank of IUPRED predicted disorder) accounted for by various combinations of predictors fitted in a linear regression model.
(PDF)

Table S4 Correlation of the IUPRED short method used in the main survey with other predictive methods (see text).
(PDF)

Table S5 Relationship between disorder and genome size within each family (like main Table 2, with interquartile ranges/minima/maxima indicating the spread of disorder within each family).
(PDF)

Table S6 Comparison of disorder of 17 viral proteins calculated by DISPROT and IUPred.
(PDF)

Table S7 Representative list illustrating the most disordered protein of greater than 200 residues from each viral family shown (excluding hypothetical proteins).
(PDF)

Results S1 **Supplementary results.**
(PDF)
