# Supplementary figures and images for: Marked Variability in the Extent of Protein Disorder within and between Viral Families
Source: PLoS One. 2013 Apr 19;8(4):e60724. doi: 10.1371/journal.pone.0060724 (PMC3631256; doi:10.1371/journal.pone.0060724)

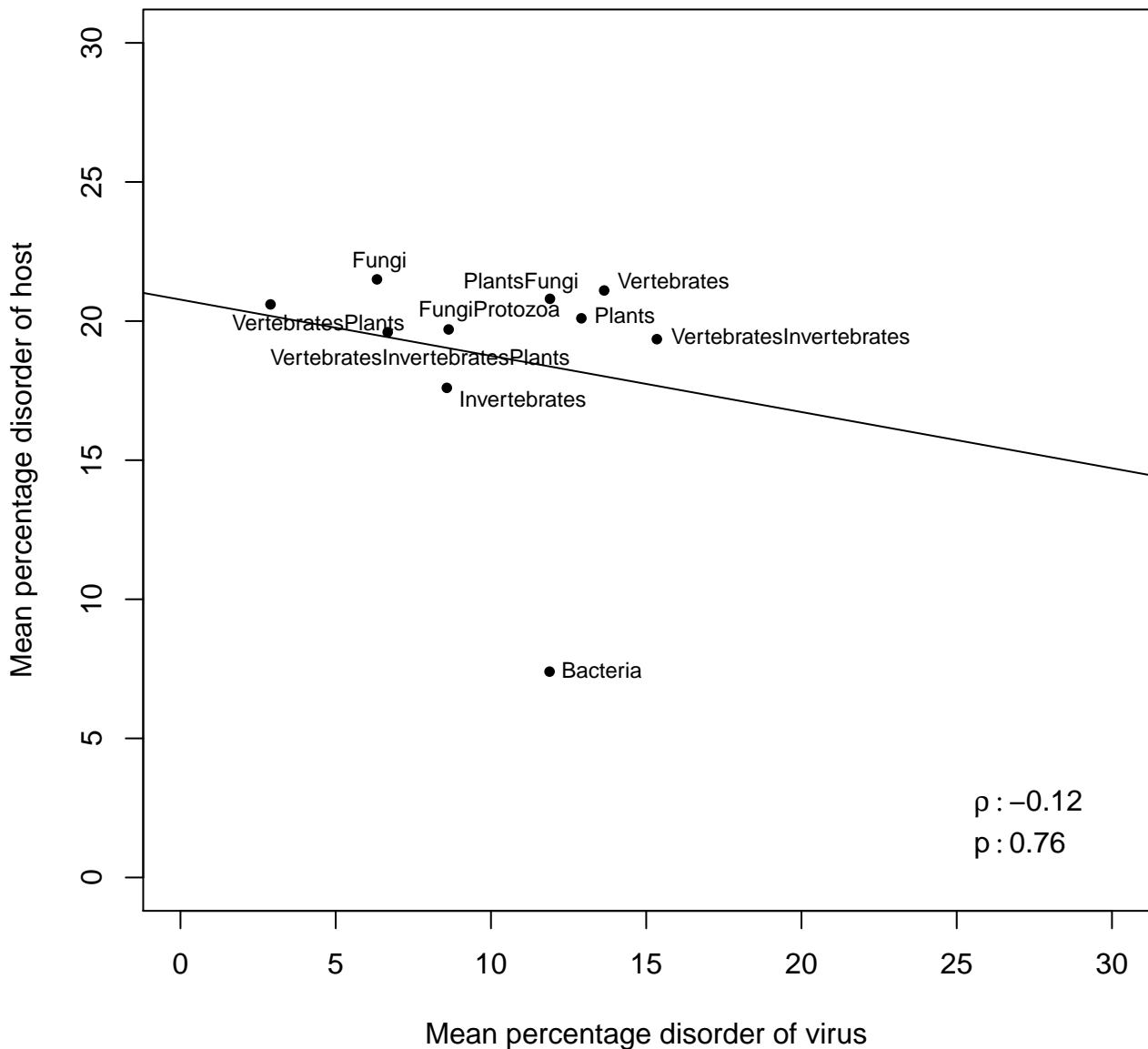

Supplement: Figure S12 — Relationship between viral and host disorder (see Table S4). (PDF) [file pone.0060724.s012.pdf]

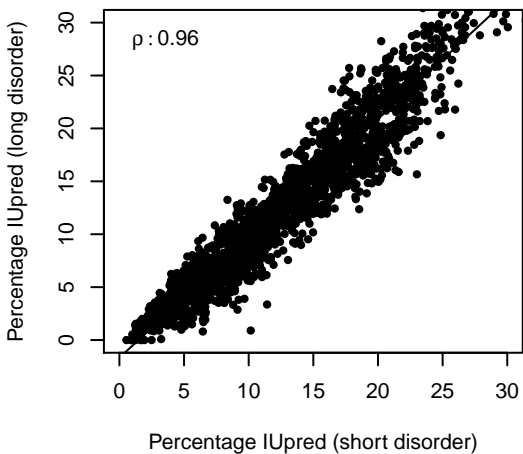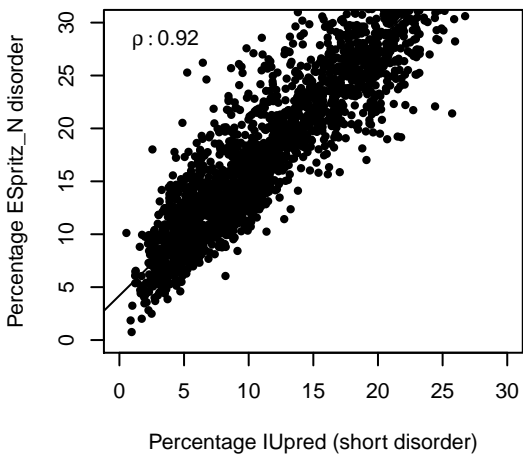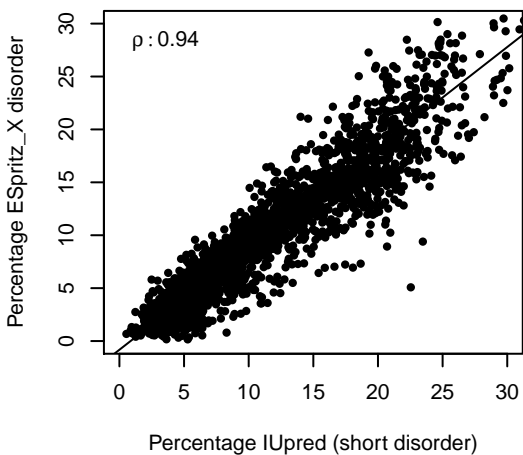

Supplement: Figure S13 — Comparison of different methods of disorder prediction for each virus in the dataset. (PDF) [file pone.0060724.s013.pdf]

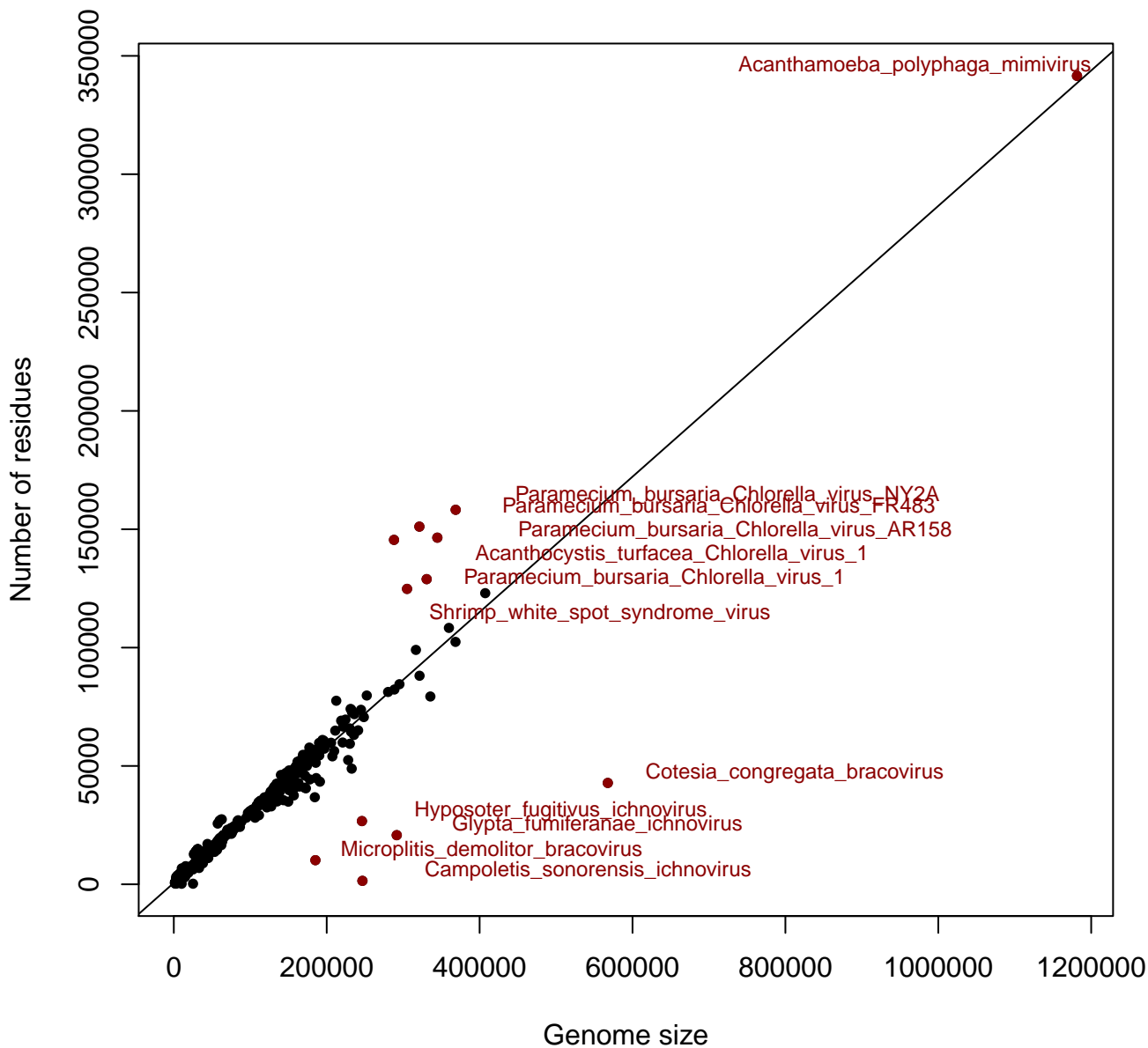

Supplement: Figure S14 — Predicted number of amino acid residues versus genome size, to highlight potential annotation errors in the dataset. (PDF) [file pone.0060724.s014.pdf]
