# Supplementary material for: Marked Variability in the Extent of Protein Disorder within and between Viral Families
Source: PLoS One. 2013 Apr 19;8(4):e60724. doi: 10.1371/journal.pone.0060724 (PMC3631256; doi:10.1371/journal.pone.0060724)
Supplement: Table S1 — Intrinsic disorder in viral genomes classified by the hosts they interact with. (PDF) [file pone.0060724.s015.pdf]

**Table S1. Intrinsic disorder in viral genomes classified by the hosts they interact with.**

| ***Host*** | $N^a$ | $S^b$ (Kbp) | | $D^c$ (%) | | Correlation[d] | | $R^2_B$ | $R^2_{BS}$ | $p^e$ |
|---|---|---|---|---|---|---|---|---|---|---|
| | | **μ** | **σ** | **μ** | **σ** | **ρ** | **p** | | | |
| Plants | 638 | 6.65 | 3.50 | 12.91 | 7.05 | -0.73 | **$2.20 \times 10^{-16}$** | 0.27 | 0.61 | **$<2 \times 10^{-16}$** |
| Vertebrates | 590 | 22.85 | 42.11 | 13.65 | 8.76 | -0.35 | **$2.20 \times 10^{-16}$** | 0.21 | 0.21 | 0.30 |
| Vertebrates/Invertebrates | 108 | 61.47 | 85.63 | 15.34 | 6.93 | -0.77 | **$2.20 \times 10^{-16}$** | 0.53 | 0.74 | **$2.09 \times 10^{-15}$** |
| Invertebrates | 90 | 109.09 | 86.58 | 8.58 | 4.25 | 0.55 | **$2.97 \times 10^{-8}$** | 0.17 | 0.40 | **$1.75 \times 10^{-7}$** |
| Bacteria | 108 | 37.41 | 17.85 | 11.89 | 3.39 | 0.44 | **$2.34 \times 10^{-6}$** | 0.19 | 0.41 | **$7.26 \times 10^{-9}$** |
| Bacteria, Archaea | 346 | 65.88 | 49.05 | 12.36 | 3.95 | 0.19 | **$3.22 \times 10^{-4}$** | 0.59 | 0.60 | 0.04 |
| Plants, Fungi | 71 | 6.54 | 2.86 | 11.90 | 4.80 | 0.31 | $8.81 \times 10^{-3}$ | 0.32 | 0.33 | 0.17 |
| Fungi | 19 | 6.23 | 4.40 | 6.33 | 5.59 | 0.57 | 0.01 | 0.65 | 0.66 | 0.32 |
| Archaea | 26 | 29.35 | 15.11 | 6.32 | 2.00 | -0.37 | 0.06 | -0.02 | -0.05 | 0.57 |
| Fungi, Protozoa | 29 | 5.67 | 1.72 | 8.64 | 4.31 | 0.23 | 0.23 | 0.39 | 0.36 | 0.89 |
| Vertebrates/Invertebrates, Plants | 39 | 23.52 | 5.87 | 6.23 | 1.49 | 0.16 | 0.34 | 0.10 | 0.09 | 0.44 |
| Bacteria, Mycoplasmas | 26 | 7.29 | 1.08 | 10.43 | 4.19 | -0.09 | 0.65 | 0.60 | 0.58 | 1.00 |
| Vertebrates, Invertebrates, Plants | 27 | 12.42 | 1.21 | 7.32 | 1.34 | -0.08 | 0.69 | -0.01 | 0.03 | 0.17 |
| Vertebrates, Plants | 25 | 13.98 | 2.70 | 2.90 | 1.22 | 0.06 | 0.77 | -0.02 | 0.40 | **$7.01 \times 10^{-4}$** |
| Bacteria, Spiroplasmas | 15 | 5.12 | 0.65 | 13.51 | 4.76 | 0.05 | 0.87 | 0.75 | 0.87 | $6.90 \times 10^{-3}$ |

[a]Number of genomes in the family.

[b]Genome size; μ and σ represent the mean and standard deviation respectively.

[c]Percent protein disorder as defined in Materials & Methods.

[d]Correlation between disorder and genome size; ρ and p represent coefficient of correlation and probability respectively.

[e]Probability that genome size is a significant predictor of disorder, from an analysis of variance model including the composition of the four bases as covariates. Significant probabilities after Bonferroni correction are marked in bold.
