# Supplementary material for: Marked Variability in the Extent of Protein Disorder within and between Viral Families
Source: PLoS One. 2013 Apr 19;8(4):e60724. doi: 10.1371/journal.pone.0060724 (PMC3631256; doi:10.1371/journal.pone.0060724)
Supplement: Table S2 — Variance in genome size explained by base composition. (PDF) [file pone.0060724.s016.pdf]

Table S2. Variance in genome size explained by base composition.

Family	***N*** [a]	***S (Kbp)*** [b]		***R^2***	***p*** [c]
		**μ**	**σ**		
Microviridae	15	5.12	0.65	0.76	**2.63×10^{-4}**
Caliciviridae	21	7.72	0.54	0.74	**2.77×10^{-5}**
Togaviridae	17	11.52	0.49	0.72	**5.34×10^{-4}**
Secoviridae	32	11.19	1.61	0.71	**8.04×10^{-8}**
Nodaviridae	12	4.49	0.08	0.66	0.02
Parvoviridae	53	5.09	0.55	0.58	**1.65×10^{-9}**
Retroviridae	57	8.45	2.39	0.54	**3.41×10^{-9}**
Anelloviridae	36	3.26	0.54	0.54	**1.02×10^{-5}**
Virgaviridae	38	8.11	2.17	0.53	**2.57×10^{-6}**
Rhabdoviridae	27	12.42	1.21	0.52	**4.12×10^{-4}**
Bunyaviridae	25	13.98	2.70	0.39	3.46×10^{-3}
Paramyxoviridae	33	15.86	1.28	0.34	1.60×10^{-3}
Tymoviridae	21	6.42	0.37	0.33	0.03
Picornaviridae	56	7.67	0.48	0.33	**6.03×10^{-5}**
Arenaviridae	26	10.52	0.15	0.27	0.03
Baculoviridae	53	131.86	21.97	0.26	**8.91×10^{-4}**
Betaflexiviridae	46	8.28	0.61	0.26	2.44×10^{-3}
Dicistroviridae	14	9.29	0.57	0.26	0.16
Myoviridae	102	109.18	70.18	0.24	**4.46×10^{-6}**
Potyviridae	79	9.83	0.41	0.21	**2.08×10^{-4}**
Coronaviridae	52	29.30	1.31	0.21	4.11×10^{-3}
Reoviridae	39	23.52	5.87	0.20	0.02
Siphoviridae	244	47.79	15.91	0.19	**7.06×10^{-11}**
Tombusviridae	43	4.27	0.46	0.16	0.03
Podoviridae	91	42.59	14.18	0.15	**9.72×10^{-4}**
Poxviridae	27	185.60	51.15	0.15	0.11
Luteoviridae	22	5.74	0.15	0.13	0.18
Partitiviridae	25	4.25	0.68	0.10	0.20
Flaviviridae	52	10.96	1.76	0.08	0.09
Alphaflexiviridae	40	6.80	0.81	0.07	0.17

Papillomaviridae	96	7.67	0.33	0.06	0.04
Herpesviridae	42	163.97	43.17	0.03	0.27
Geminiviridae	254	3.59	1.20	0.03	0.02
Caulimoviridae	36	7.82	0.44	0.03	0.31
Totiviridae	29	5.67	1.72	0.03	0.34
Adenoviridae	26	34.85	8.34	0.00	0.40
Bromoviridae	29	8.43	0.27	0.00	0.44
Polyomaviridae	21	5.14	0.17	-0.08	0.65
Inoviridae	26	7.29	1.08	-0.10	0.87
Closteroviridae	25	16.31	1.38	-0.12	0.84
Circoviridae	16	2.05	0.41	-0.28	0.95

[a]Number of genomes in the family.

[b]Genome size; μ and σ represent the mean and standard deviation respectively.

[c]Probability that genome size is a significant predictor of disorder, from an analysis of variance model including the composition of the four bases as covariates. Significant probabilities after Bonferroni correction are marked in bold.
