# Supplementary material for: Marked Variability in the Extent of Protein Disorder within and between Viral Families
Source: PLoS One. 2013 Apr 19;8(4):e60724. doi: 10.1371/journal.pone.0060724 (PMC3631256; doi:10.1371/journal.pone.0060724)
Supplement: Table S3 — Percentage of variance in disorder (Adjusted R2 for the rank of IUPRED predicted disorder) accounted for by various combinations of predictors fitted in a linear regression model. (PDF) [file pone.0060724.s017.pdf]

Table S3: Percentage of variance in disorder (Adjusted R^2 for the rank of IUPRED predicted disorder) accounted for by various combinations of predictors fitted in a linear regression model.

Predictor(s)[a]	*Adjusted R^2*
A	0.04
T	0.25
G	0.03
C	0.22
A, T, G, C,	0.35
A, T, G, C, S	0.41
S	0.09
Type	0.36
H	0.10
F	0.74
Type, H, F	0.74
Type, A, T, G, C	0.59
H, A, T, G, C	0.42
F, A, T, G, C	0.83
F, S	0.74
F, A, T, G, C, S	0.83

[a]A: % Adenine; T: % Thymine; G: % Guanine; C: % Cytosine. S: rank of genome size (ranked in order to correct for markedly non-normal distribution). Type: viral type (see Table 1); H: viral host (see Table S1); F: viral family (see Table 2).
