# Supplementary material for: Marked Variability in the Extent of Protein Disorder within and between Viral Families
Source: PLoS One. 2013 Apr 19;8(4):e60724. doi: 10.1371/journal.pone.0060724 (PMC3631256; doi:10.1371/journal.pone.0060724)
Supplement: Table S4 — Correlation of the IUPRED short method used in the main survey with other predictive methods (see text). (PDF) [file pone.0060724.s018.pdf]

Table S4. Correlation of the IUPRED short method used in the main survey with other predictive methods (see text).

**Method**	**Mean**	**Spearman's rho**
IUPRED short	12.4	1.00
IUPRED long	12.3	0.96
ESPRITZ N	19.1	0.92
ESPRITZ X	11.0	0.94
IUPRED short, regions	21.8*	0.50
ESPRITZ N, regions	31.6*	0.62
ESPRITZ X, regions	24.1*	0.68

***** percentage of proteins in a virus that have at least one long disordered region of 30 or more residues.
