# Supplementary material for: Marked Variability in the Extent of Protein Disorder within and between Viral Families
Source: PLoS One. 2013 Apr 19;8(4):e60724. doi: 10.1371/journal.pone.0060724 (PMC3631256; doi:10.1371/journal.pone.0060724)
Supplement: Table S5 — Relationship between disorder and genome size within each family (like main Table 2, with interquartile ranges/minima/maxima indicating the spread of disorder within each family). (PDF) [file pone.0060724.s019.pdf]

Table S5: Relationship between disorder and genome size within each family (like main Table 2, with interquartile ranges/minima/maxima indicating the spread of disorder within each family).

Family	***N*[a]**	***S*[b] *(Kbp)***		***D*[c] *(%)***						***Correlation*[d]**		***R^2_B***	***R^2_{BS}***	***p*[e]**
		**μ**	**σ**	**μ**	**σ**	**interquartile**		**Min**	**Max**	**ρ**	**P**			
Geminiviridae	254	3.59	1.20	18.57	3.13	16.6	20.7	9.3	26.2	-0.38	**5.0×10^{-10}**	0.10	0.22	**2.51×10^{-9}**
Virgaviridae	38	8.11	2.17	4.93	2.45	3.2	6.0	1.9	11.5	0.78	**9.9×10^{-9}**	0.13	0.64	**3.70×10^{-8}**
Alphaflexiviridae	40	6.80	0.81	13.94	4.58	10.3	17.4	3.8	24.5	0.57	**1.7×10^{-4}**	0.35	0.71	**1.16×10^{-7}**
Podoviridae	91	42.59	14.18	12.47	2.93	10.6	13.7	6.0	20.9	0.29	5.9×10^{-3}	0.24	0.35	**2.29×10^{-4}**
Bromoviridae	29	8.43	0.27	8.52	2.01	7.2	9.6	5.6	12.7	0.57	1.5×10^{-3}	0.30	0.59	**2.73×10^{-4}**
Bunyaviridae	25	13.98	2.70	2.90	1.22	2.3	3.6	1.0	6.6	0.06	0.77	-0.02	0.40	**7.01×10^{-4}**
Myoviridae	102	109.18	70.18	11.42	3.91	8.8	12.7	4.7	29.0	-0.29	3.1×10^{-3}	0.57	0.61	**1.17×10^{-3}**
Microviridae	15	5.12	0.65	13.51	4.76	11.8	13.6	8.1	29.9	0.05	0.87	0.75	0.87	6.90×10^{-3}
Poxviridae	27	185.60	51.15	5.63	2.75	3.6	5.9	2.7	13.9	0.11	0.57	0.83	0.87	0.01
Paramyxoviridae	33	15.86	1.28	12.37	2.97	9.5	14.3	8.2	18.3	0.41	0.02	0.14	0.29	0.01
Potyviridae	79	9.83	0.41	5.51	1.54	4.4	6.4	2.7	10.4	0.06	0.59	0.22	0.27	0.01
Secoviridae	32	11.19	1.61	4.16	2.08	2.7	5.4	1.2	9.8	0.71	**4.7×10^{-6}**	0.37	0.48	0.01
Coronaviridae	52	29.30	1.31	3.68	1.33	3.1	4.2	1.8	8.4	0.29	0.04	0.32	0.39	0.02
Picornaviridae	56	7.67	0.48	6.53	2.51	4.5	8.5	2.3	11.5	0.59	**2.2×10^{-6}**	0.72	0.74	0.02
Parvoviridae	53	5.09	0.55	19.96	3.66	17.8	22.5	13.1	32.0	-0.42	1.7×10^{-3}	0.30	0.35	0.03
Tombusviridae	43	4.27	0.46	10.29	3.12	8.2	12.7	4.4	17.1	-0.26	0.10	0.32	0.38	0.04
Papillomaviridae	96	7.67	0.33	21.17	4.02	18.7	23.3	10.8	30.1	-0.14	0.18	0.46	0.47	0.09
Closteroviridae	25	16.31	1.38	5.85	1.98	4.6	7.3	2.3	10.5	-0.22	0.29	0.17	0.24	0.10
Luteoviridae	22	5.74	0.15	23.08	2.36	20.9	24.9	19.8	29.0	0.43	0.04	0.34	0.41	0.11
Nodaviridae	12	4.49	0.08	18.51	2.33	17.0	18.8	15.4	22.9	0.11	0.74	0.74	0.80	0.11
Polyomaviridae	21	5.14	0.17	18.62	4.80	15.8	20.6	10.5	30.5	0.40	0.07	0.69	0.71	0.16
Rhabdoviridae	27	12.42	1.21	7.32	1.34	6.4	8.0	4.3	11.4	-0.08	0.69	-0.01	0.03	0.17

Adenoviridae	26	34.85	8.34	16.14	4.59	14.0	17.8	8.4	25.4	0.60	1.5×10^{-3}	0.74	0.75	0.17
Partitiviridae	25	4.25	0.68	9.88	3.74	7.9	11.2	3.0	11.7	0.20	0.35	0.32	0.34	0.23
Siphoviridae	244	47.79	15.91	12.75	3.91	9.7	15.2	6.7	28.2	0.55	**2.2×10^{-16}**	0.62	0.62	0.23
Togaviridae	17	11.52	0.49	8.78	3.13	7.4	9.2	4.4	17.4	0.36	0.15	0.78	0.79	0.26
Flaviviridae	52	10.96	1.76	5.23	1.84	4.1	6.1	2.2	10.0	-0.15	0.28	0.71	0.71	0.30
Retroviridae	57	8.45	2.39	17.94	7.01	13.1	20.6	2.5	38.9	-0.17	0.21	0.24	0.24	0.33
Arenaviridae	26	10.52	0.15	3.58	0.80	3.0	4.2	2.4	5.9	-0.09	0.67	0.12	0.12	0.35
Reoviridae	39	23.52	5.87	6.23	1.49	5.1	7.0	3.3	9.8	0.16	0.34	0.10	0.09	0.44
Baculoviridae	53	131.86	21.97	8.14	1.50	7.0	8.9	5.4	12.0	0.47	**4.3×10^{-4}**	0.57	0.56	0.48
Herpesviridae	42	163.97	43.17	17.87	5.40	13.7	22.1	7.6	29.7	0.48	1.4×10^{-3}	0.73	0.73	0.49
Tymoviridae	21	6.42	0.37	22.83	7.03	19.0	28.1	9.8	35.3	0.26	0.25	0.50	0.48	0.53
Betaflexiviridae	46	8.28	0.61	6.74	2.06	5.2	8.0	3.0	11.5	-0.17	0.26	0.11	0.09	0.63
Caulimoviridae	36	7.82	0.44	14.55	4.21	11.0	18.2	7.2	22.4	0.08	0.66	0.20	0.17	0.71
Anelloviridae	36	3.26	0.54	29.50	8.31	22.8	35.2	15.6	45.2	0.26	0.13	0.38	0.36	0.72
Caliciviridae	21	7.72	0.54	8.33	2.94	6.2	9.4	4.0	17.5	-0.17	0.45	0.15	0.10	0.73
Dicistroviridae	14	9.29	0.57	5.17	1.74	3.8	6.0	2.4	9.5	0.20	0.48	0.66	0.63	0.76
Totiviridae	29	5.67	1.72	8.64	4.31	5.8	10.8	2.6	17.5	0.23	0.22	0.39	0.36	0.89
Circoviridae	16	2.05	0.41	12.75	8.86	8.2	13.6	4.2	13.9	0.07	0.79	0.58	0.53	0.92
Inoviridae	26	7.29	1.08	10.43	4.19	7.2	12.7	3.7	18.3	-0.09	0.65	0.60	0.58	1.00
