# Supplementary material for: Marked Variability in the Extent of Protein Disorder within and between Viral Families
Source: PLoS One. 2013 Apr 19;8(4):e60724. doi: 10.1371/journal.pone.0060724 (PMC3631256; doi:10.1371/journal.pone.0060724)
Supplement: Table S6 — Comparison of disorder of 17 viral proteins calculated by DISPROT and IUPred. (PDF) [file pone.0060724.s020.pdf]

Table S6: Comparison of disorder of 17 viral proteins calculated by DISPROT and IUPred.

DISPROT ID	Name	Organism	Length	GC	D (%)		Diff.
					Disprot	Iupred	
DP00560	Genome Polyprotein	Turnip mosaic virus	3163	56	6	6.35	0.35
DP00087	Alpha trans-inducing protein	Human herpesvirus 2 (strain HG52) (Human herpes simplex virus 2)	490	70	27	25.51	1.49
DP00066	Structural polyprotein	Sindbis virus (subtype Ockelbo / strain Edsbyn 82-5)	264	51	40	42.80	2.80
DP00431	polyprotein	Sesbania mosaic virus	962	50	8	12.47	4.47
DP00160	Measles virus nucleocapsid protein	Measles virus	525	47	24	28.57	4.57
DP00189	Nef protein	Human immunodeficiency virus type 1 (isolate 12)	206	42	21	14.56	6.44
DP00424	REV protein	Human immunodeficiency virus 1	116	42	47	53.45	6.45
DP00064	Coat protein [Precursor]	Southern bean mosaic virus	260	50	25	17.69	7.31
DP00447	Phosphoprotein	Human respiratory syncytial virus (strain Long)	241	34	58	49.79	8.21
DP00585	Flavivirus E Glycoprotein	Dengue virus type 2	395	46	1	11.14	10.14
DP00182	Coat protein VP1	Simian virus 40	364	41	15	31.59	16.59
DP00101	HIV Type 1 p6 Protein	Human immunodeficiency virus type 1	499	42	7	27.45	20.45
DP00003	Adenovirus ssDNA binding protein	Human adenovirus type 5	529	55	10	31.95	21.95
DP00048	Nef protein	Human immunodeficiency virus type 1 (BRU isolate)	206	42	52	19.42	32.58
DP00024	E7 protein	Human papillomavirus type 16	98	37	100	41.84	58.16
DP00148	NCp7	Human immunodeficiency virus 1	71	42	100	21.13	78.87
DP00542	VPg	Potato virus Y	185	42	100	12.97	87.03
