# Supplementary material for: Marked Variability in the Extent of Protein Disorder within and between Viral Families
Source: PLoS One. 2013 Apr 19;8(4):e60724. doi: 10.1371/journal.pone.0060724 (PMC3631256; doi:10.1371/journal.pone.0060724)
Supplement: Table S7 — Representative list illustrating the most disordered protein of greater than 200 residues from each viral family shown (excluding hypothetical proteins). (PDF) [file pone.0060724.s021.pdf]

Table S7. Representative list illustrating the most disordered protein of greater than 200 residues from each viral family shown (excluding hypothetical proteins).

(A) Genome size more than 10kb.

Family	Virus	Accession	Product	Length	%Disorder
Herpesviridae	Callitrichine herpesvirus 3	NP_733919	C4	254	100.00
Alloherpesviridae	Anguillid herpesvirus 1	YP_003358141	ORF2L	345	95.36
Nimaviridae	Shrimp white spot syndrome virus	NP_477701	wsv179	221	93.67
Phycodnaviridae	Ectocarpus siliculosus virus 1	NP_077542	EsV-1-57	358	88.27
Podoviridae	Burkholderia phage BcepIL02	YP_002922702	gp30	258	86.43
Paramyxoviridae	Human parainfluenza virus 3	NP_599250	D protein	373	86.33
Polydnaviridae	Hyposoter fugitivus ichnovirus	YP_001031291	c17.1	352	84.38
Siphoviridae	Mycobacterium phage Pukovnik	YP_001994825	gp7	356	83.71
Myoviridae	Pseudomonas phage LMA2	YP_002154300	putative structural protein	297	79.46
Adenoviridae	Porcine adenovirus A	YP_009215	22K	225	78.67
Baculoviridae	Lymantria dispar MNPV	NP_047640	mucin-like protein	1029	75.90
Poxviridae	Cowpox virus	NP_619919	CPXV136 protein	295	73.22
Reoviridae	Rice gall dwarf virus	YP_001111377	RNA-binding protein 12	206	68.93
Ascoviridae	Spodoptera frugiperda ascovirus 1a	YP_762403	64.6 kDa	565	68.32
Coronaviridae	SARS coronavirus	NP_828858	nucleocapsid protein	422	67.77
Tectiviridae	Bacillus phage AP50	YP_002302524	virion assembly protein	235	62.98
Malacoherpesviridae	Ostreid herpesvirus 1	YP_024574	ORF29	201	60.20
Asfarviridae	African swine fever virus	NP_042837	pDP238L	238	57.14
Orthomyxoviridae	Infectious salmon anemia virus	YP_145796	P6	234	54.70
Rhabdoviridae	Tupaia virus	YP_238530	protein C	221	52.49

(B) Genome size less than 10kb.

Family	Virus	Accession	Product	Length	% disorder
Tymoviridae	Turnip yellow mosaic virus	NP_663296	overlapping protein/movement protein	628	91.56
Papillomaviridae	Human papillomavirus type 5	NP_041369	E4	245	86.12
Anelloviridae	Torque teno virus 3	YP_003587865	ORF2/2	281	77.22
Polyomaviridae	Finch polyomavirus	YP_529830	putative ORF-X	205	68.29
Parvoviridae	Human bocavirus	YP_338087	NP-1	219	55.25
Alphaflexiviridae	Shallot virus X	NP_620652	coat protein	262	53.82
