# Supplementary material for: Marked Variability in the Extent of Protein Disorder within and between Viral Families
Source: PLoS One. 2013 Apr 19;8(4):e60724. doi: 10.1371/journal.pone.0060724 (PMC3631256; doi:10.1371/journal.pone.0060724)
Supplement: Results S1 — Supplementary results. (PDF) [file pone.0060724.s022.pdf]

SUPPORTING INFORMATION

RESULTS

GC content is a strong predictor of intrinsic disorder

Disordered amino acids have higher frequencies of certain bases and viruses show strong biases in base composition. Therefore it is important to check whether base composition is correlated with viral genome size. Any such correlations could potentially explain the observed association (see main text) between disorder and genome size. While in host genomes, base composition can be represented by G+C content, the asymmetric replication of viral strands frequently gives rise to biases in base composition in which G and C are not at equal frequencies. Intrinsically disordered proteins are less complex and are dominated by certain residues (E, K, R, G, Q, S, P, A) [1,2,3,4]. Mutational pressure has been the dominant evolution driving force in determining the different codon usage preferences in ssRNA viruses [5]. For example, coding sequences of all negative-stranded RNA viruses are biased toward high A in the coding sequences and high T in genomes suggesting that RNA viruses with different genome polarity are under different mutational pressure [6].

We investigated whether intrinsic disorder is determined by GC content in these viral sequences. Indeed, intrinsic disorder shows an overall significant positive correlation ($\rho = 0.36$, $p < 2.2 \times 10^{-16}$) with the GC content; however, it is only a partial determinant (the proportion of variance explained, $R^2 = 0.13$). Within families too, this trend is seen i.e. all families show a positive correlation of intrinsic disorder with GC content, with the exceptions of *Tombusviridae*, *Betaflexiviridae*, *Circoviridae* and *Nodaviridae* which show negative correlations; however none of these are significant ($p > 0.05$).

GC content and experimentally observed disorder

We wished to check if the relationship between GC content and predicted disorder in viral sequences is reflected in a similar relationship between GC content and experimentally determined disorder. A number of viral proteins with an appreciable extent of disorder have been structurally investigated. We investigated 17 viral proteins from the DISPROT [7] database of disordered proteins of known structure (Table S6). We anticipated that there would be a higher GC content among those viral proteins for which a greater proportion of the residues are observed to be disordered. We noted that IUPred strongly under-predicted the extent of disorder in the shorter proteins (Table S3). Among the longer proteins, the one

whose gene had the highest GC content (HHV2 alpha trans-inducing protein), had an IUPred prediction that matched the DISPROT observation exactly, suggesting that GC content does not necessarily bias IUPred predictions upwards. Selecting the nine proteins that are more than 250 residues long, there is a positive correlation between observed disorder and GC content (Pearson's correlation coefficient of 0.38), but the sample size is too small to draw any general inference. However, we note that this correlation is similar to that seen for predicted disorder. Thus, we have no reason to believe that IUPred is over-predicting disorder in GC rich proteins, since the observed disorder in this small dataset of larger proteins shows a similar level of correlation.

Base composition is a partial predictor of genome size in families

We explored the individual correlations between genome size and base composition. Genome size in viral genomes is positively correlated with the proportion of A ($\rho = 0.09$) and G ($\rho = 0.0001$) and negatively correlated with T ($\rho = -0.068$) and C ($\rho = -0.064$). However, the p-value associated with G was not significant ($p = 0.99$). We did a multiple linear regression to determine the relationship between genome size and base composition. Overall only 0.3% of the overall variance in genome size could be explained by base composition. However, within 19 families with ten or more viral genomes, between 15% (*Podoviridae*) and 76% (*Microviridae*) of the variance in genome size could be explained by base composition ($p < 0.01$; Table 2). Some of the families such as *Microviridae* (76%), *Calciviridae* (74%), *Togaviridae* (72%), *Secoviridae* (71%), *Nodaviridae* (66%), *Parvoviridae* (58%), *Retroviridae* (54%), *Anelloviridae* (54%), *Virgaviridae* (53%) and *Rhabdoviridae* (52%) have more than 50% variance in genome size associated with base composition. *Poxviridae* and *Herpesviridae* have bigger genomes but the variance explained by base composition is 15% and 3% respectively. *Geminiviridae* (N = 254) is the biggest family of viruses but only 3% of variance in genome size is explained by base composition. Thus, we conclude that base composition could be a partial predictor of genome size within viral families. Therefore, since disorder is also related to base composition, we need to account for this when investigating the relationship between disorder and genome size.

Smaller genomes with greater disorder often have more overlapping genes in ssRNAp and the ssDNA viruses.

Disordered regions are often found in overlapping regions of viral proteins [8]. This may arise in part because the constraints on out of frame proteins alter the amino acid preferences, in part because many overlapping proteins are short. Shorter proteins tend to be more disordered, and may be in part because overlapping proteins may be accessory proteins, without a key conserved structural role. The negative

correlation seen for most viral types may in some instances relate to family specific effects within the type. Among the ssRNAp, the negative correlation can largely be accounted for by the large size and low disorder of the *Coronaviridae* (with large non-segmented RNA genomes; Figure S4), contrasted with the high disorder of a subset of the smaller families, primarily the *Luteoviridae*, the *Tymoviridae*, and the *Alphaflexiviridae* (Figure S5). In terms of understanding the biology of these proteins, the latter three families have a greater degree of overlap in their genomes, in contrast with the *Coronaviridae*. Overlapping proteins may in some circumstances be more disordered [8]. Among the ssDNA, the *Annelloviridae* are short and disordered, while the *Nanoviridae* and the *Inoviridae* are longer and ordered (Figure S6). For this group, as for the ssRNAp, the short and disordered families appear to have more overlapping open reading frames (Figure S7). There is a very large number of dsDNA viruses, but the negative correlation in part reflects a very small genome size and high disorder for the *Papillomaviridae* (Figure S8). Again these viruses exhibit a large degree of viral gene overlap. Among the dsDNA-RT, the shorter *Hepadnaviridae* have more disorder than the longer *Caulimoviridae (Figure S9).* Again, it is striking that the *Hepadnaviridae* display more overlapping open reading frames than any other.

Greater disorder among larger genomes of ssRNAn viruses unlikely to reflect differences in extent of overlapping genes.

We were interested as to why ssRNAn viruses should show an opposite (positive) correlation between genome size and disorder compared to the relationship seen among the other viral types. One feature that distinguishes this type is that it has the lowest mean disorder (μ_D = 7.4%; Table 1). A second feature of this type is that it has a low variability in genome size (Table 1). However, there could be other explanations. One question is whether the individual families within this type show very different patterns. While indeed, the correlation could be explained to some degree by the fact that the *Paramyxoviridae* have larger genomes and more disorder than the *Bunyaviridae, Rhabdoviridiae* and *Arenaviridae*, there was still a positive correlation among the *Paramyxoviridae* (Table 2; Figure S10). In this type, overlapping sequences are found within both the more disordered *Paramyxoviridae* and in the less disordered smaller *Bunyaviridae* (Figure S11). Thus, the trend is likely to reflect other aspects of viral function. This may relate to additional proteins, or in the case of *Filoviridae* to the addition of a disordered region to the NCAP protein [9], which may partly explain the observation.

Thus, overall we see a general pattern emerging within each major type. Typically, the more disordered viral proteomes have more overlapping proteins. Strikingly, in one viral type, the larger genomes are more disordered, but there is no relationship between disorder and overlapping genes. If we put this one unusual type to one side, we are left with a general trend that the viruses with more disorder have smaller genomes with more overlapping genes. There are two potential explanations for this association. The first is that the overlapping segments are themselves more disordered. The second is that the solution of

overlapping proteins, and of more disorder, are both independent responses to the problem of how to fit more coding functionality into the viral proteome encoded by a spatially constrained genome.

To address this, we plotted the predicted disorder across some representative proteins containing overlapping segments. For *Hepatitis B*, some of the overlapping gene regions of protein P appear disordered, either in protein P, or in protein S. A more systematic analysis of this relationship is complicated, since many overlapping proteins are very short, and very short proteins are often very disordered simply because they are too short to support an ordered structure. Thus, it is difficult to determine whether the overlapping regions have a much higher disorder than expected, given their short length, compared with other similarly short regions.

Considering cleaved polypeptides produced similar results

We also investigated the effect of polyproteins that are cleaved to produce a number of polypeptides. A total of 545 polyproteins were present in the dataset. Percent disorder for each polyprotein (D_p) was compared with the resultant disorder (D_c) of all corresponding cleaved polypeptides (Figure S3). The percent disorder values obtained from both methods were significantly correlated ($\rho = 0.85$, $p < 2.2 \times 10^{-16}$). When we incorporated these to the prediction of percent disorder for the whole genome, we found that both methods produced almost similar values of percent disorder ($\rho = 0.87$, $p < 2.2 \times 10^{-16}$; Figure S3).

We repeated the complete analyses by substituting percent disorder with the new percent disorder obtained from cleaved peptides and found similar results. Thus, while for individual proteins, investigators need to consider carefully whether they are interested in the disorder in the precursor polyprotein, or as is more usually of interest, in the mature post-cleavage products, it is important to ensure that the predictions are carried out on the appropriate state, since disorder is greater at protein termini. However, for this overall survey it is unlikely that the true cleavage states of all protein products are known. Our overall analysis will tend towards under-predicting the disorder of proteins found within poloyproteins, since in general incomplete viral genome analysis under-predicts cleavage. However, the overall analysis appears insensitive to the minor biases introduced by this under-prediction.

Effect of alternative approaches to estimating disorder

We noted that short and long disorder predictions from IUPRED gave highly correlated results (Supplementary Figure S13). There was a reasonable correlation of predictions using an alternative method, E-spritz[10], which is a machine learning predictor trained on known datasets, in contrast to the biophysical prediction of IUPRED (Supplementary Fig. S13). The results were broadly similar. For example, with ESPRITZ_X, base composition, family and genome size accounted for 72% of variance,

with base composition alone accounting for 28%, and family alone for 64%. These contributions are broadly similar to the trends seen with IUPRED (Supplementary Table S3), suggesting that the precise method of prediction does not greatly alter the conclusions drawn.

While we focused on predictions for individual residues, it must be noted that disorder predictions are strongly correlated with the predictions for adjacent residues, so that much of these residues fall into regions of more extended disorder. Supplementary Table S4 indicates that estimating a viral proteome's disorder propensity based on regions rather than on residues generated results that were broadly correlated. We opted to focus on residue predictions, to avoid the increase in sampling variance created by surveying the presence or absence of larger regions across very short proteins and smaller proteomes.

Which proteins display strong disorder?

We selected the most disordered proteins from each family in order to sample and illustrate typical potential roles and functional relevance. We looked among viruses longer than 10kb (all of which are dsDNA and ssRNA) at all viral proteins with more than 200 residues and with high disorder (D > 50%). Table 4a shows the most disordered protein identified in each family. These proteins are discussed further below.

C4 from *Callitrichine herpesvirus 3* is predicted to be 100% disordered. This viral protein was identified in this marmoset virus, but displayed no sequence homology to previously identified proteins [11]. This is not unusual for disordered proteins, which often evolve much faster than ordered proteins. It does present a particular challenge in elucidating the role of these proteins in viruses, since homology to ordered domains often gives insights into their functional roles. The *Porcine adenovirus* A protein 22K (DUF2890) has homologues across many adenoviruses, but the role of this disordered protein has not been established. While the herpesvirus protein with 100% predicted disorder is clearly high, the average disorder for *Herpesviridae* (Table 2) is also relatively high, at 17.9%. The observation that another protein (CPXV136) from the large *Cowpox virus* is 73% disordered is particularly striking, in the context that the mean disorder for this genome is only 5.6% (Table 2). It will be very interesting to determine whether this protein interacts with host components.

The D protein of *human parainfluenza virus* 3 is derived from an internal alternative reading frame within protein P [12], so it is possible that the level of disorder may have been affected by the coding constraints of the P gene. The 64.6kDa ascoviral protein (Table 4) shows a strong amino acid compositional bias, being highly basic [13]. This is likely to relate to the particular, and unidentified, function of this protein.

The *Lymantria dispar MNPV* mucin-like protein (putatively involved in horizontal gene transfer [14]) is one of the longer disordered proteins identified (L = 1029, D = 76%). Other long proteins included two hypothetical proteins from *Cotesia congregata bracovirus* (L = 671, D = 88%) and *Acidianus two-tailed virus* (L = 567, D = 72%) and the 64.6 kDA protein from *Spodoptera frugiperda ascovirus 1a* (L = 565, D = 68%). The function of the gp30 protein from the large *Burkholderia phage BcepIL02* has not been identified, but such a highly disordered protein may play a role in interaction with its host bacterium. Interestingly, this protein displays suggestive similarity to a protein from its host (27% identity over 162 residues to the protein BURPS1710b_2246 from *Burkholderia pseudomallei)***,** raising the possibility that this disordered protein may have been hijacked from host to phage, or vice versa. As these phages have been investigated as therapeutic treatments for bacterial infection [15,16], it may be of interest to determine the role of this disordered phage protein. Two of the predicted highly disordered proteins from larger viruses encoded collagen-like proteins (*Lymphocystis disease virus 1*, accession NP_078660; and *Acanthamoeba polyphaga mimivirus* YP_142550). If these proteins do indeed form collagen like triple helical structures, they are clearly not disordered proteins while forming that structure. The fact that the mimivirus encodes enzymes capable of hydroxylating lysine increases the likelihood that this is indeed the case, and it has been speculated that the collagen-like fibres may form part of the fibre layer surrounding the Mimivirus particle [17]. If this is the case, such proteins are not strictly disordered in their typical state, and therefore this represents an incorrect prediction of the IUPRED method, and we have accordingly omitted them from Table 4. However, it is worth considering that these proteins also perform biological functions in their monomeric disordered states.

While disordered proteins from the larger viruses discussed above may benefit from the availability of greater genomic space in which they can evolve and acquire functions, we found that the smaller viruses showed a similar range of functions for their longer disordered proteins (Table 4b). The overlapping/movement protein from *Turnip yellow mosaic virus* was particularly long and disordered, given the smaller genome size (L = 628, D = 92%). Movement proteins are encoded by many plant-infecting viruses, and are important because of their ability to allow movement from the initially infected cell to neighboring cells [18]. The E4 protein of *Human Papillomavirus* has been identified to harbor cyclin-binding motifs, supporting a role for this disordered protein in interacting with the host proteome to support genome amplification [19]. NP1 of *Bocavirus* is also important in DNA replication [20]. A similar nuclear role is implicated for human *Torque Teno Virus* ORF2/2, which has been postulated to play a role in binding nucleic acids during either transcription or replication [21,22]. ORF-X of *Finch polyomavirus* is an apparent accessory protein not present in other polyomaviruses [23], whose role is as yet unclear.

Thus, disordered proteins from both small and large genome viruses can play roles in viral structure and function, including interactions with host proteins.

REFERENCES

1. Uversky VN (2002) Natively unfolded proteins: a point where biology waits for physics. Protein Sci 11: 739-756.
2. Tompa P (2002) Intrinsically unstructured proteins. Trends Biochem Sci 27: 527-533.
3. Meszaros B, Tompa P, Simon I, Dosztanyi Z (2007) Molecular principles of the interactions of disordered proteins. J Mol Biol 372: 549-561.
4. Tran HT, Mao A, Pappu RV (2008) Role of backbone-solvent interactions in determining conformational equilibria of intrinsically disordered proteins. J Am Chem Soc 130: 7380-7392.
5. Su MW, Lin HM, Yuan HS, Chu WC (2009) Categorizing host-dependent RNA viruses by principal component analysis of their codon usage preferences. J Comput Biol 16: 1539-1547.
6. Auewarakul P (2005) Composition bias and genome polarity of RNA viruses. Virus Res 109: 33-37.
7. Sickmeier M, Hamilton JA, LeGall T, Vacic V, Cortese MS, et al. (2007) DisProt: the Database of Disordered Proteins. Nucleic Acids Res 35: D786-793.
8. Rancurel C, Khosravi M, Dunker AK, Romero PR, Karlin D (2009) Overlapping genes produce proteins with unusual sequence properties and offer insight into de novo protein creation. J Virol 83: 10719-10736.
9. Cleveland SB, Davies J, McClure MA A bioinformatics approach to the structure, function, and evolution of the nucleoprotein of the order mononegavirales. PLoS One 6: e19275.
10. Walsh I, Martin AJ, Di Domenico T, Tosatto SC ESpritz: accurate and fast prediction of protein disorder. Bioinformatics 28: 503-509.
11. Rivailler P, Cho YG, Wang F (2002) Complete genomic sequence of an Epstein-Barr virus-related herpesvirus naturally infecting a new world primate: a defining point in the evolution of oncogenic lymphocryptoviruses. J Virol 76: 12055-12068.
12. Durbin AP, McAuliffe JM, Collins PL, Murphy BR (1999) Mutations in the C, D, and V open reading frames of human parainfluenza virus type 3 attenuate replication in rodents and primates. Virology 261: 319-330.
13. Tan Y, Bideshi DK, Johnson JJ, Bigot Y, Federici BA (2009) Proteomic analysis of the Spodoptera frugiperda ascovirus 1a virion reveals 21 proteins. J Gen Virol 90: 359-365.
14. Monier A, Claverie JM, Ogata H (2007) Horizontal gene transfer and nucleotide compositional anomaly in large DNA viruses. BMC Genomics 8: 456.
15. Lynch KH, Stothard P, Dennis JJ Genomic analysis and relatedness of P2-like phages of the Burkholderia cepacia complex. BMC Genomics 11: 599.
16. Carmody LA, Gill JJ, Summer EJ, Sajjan US, Gonzalez CF, et al. Efficacy of bacteriophage therapy in a model of Burkholderia cenocepacia pulmonary infection. J Infect Dis 201: 264-271.
17. Claverie J, Abergel C, Ogata H (2009) Mimivirus. In: van Etten J, editor. Lesser Known Large DsDNA Viruses. Berlin: Springer Verlag. pp. 89-122.
18. Taliansky M, Torrance L, Kalinina NO (2008) Role of Plant Virus Movement Proteins. Plant Virology Protocols: From Viral Sequence to Protein Function. pp. 33-54.
19. Knight GL, Pugh AG, Yates E, Bell I, Wilson R, et al. A cyclin-binding motif in human papillomavirus type 18 (HPV18) E1^E4 is necessary for association with CDK-cyclin complexes and G2/M cell

cycle arrest of keratinocytes, but is not required for differentiation-dependent viral genome amplification or L1 capsid protein expression. Virology.

20. Sun Y, Chen AY, Cheng F, Guan W, Johnson FB, et al. (2009) Molecular characterization of infectious clones of the minute virus of canines reveals unique features of bocaviruses. J Virol 83: 3956-3967.
21. Tanaka Y, Primi D, Wang RY, Umemura T, Yeo AE, et al. (2001) Genomic and molecular evolutionary analysis of a newly identified infectious agent (SEN virus) and its relationship to the TT virus family. J Infect Dis 183: 359-367.
22. Mueller B, Maerz A, Doberstein K, Finsterbusch T, Mankertz A (2008) Gene expression of the human Torque Teno Virus isolate P/1C1. Virology 381: 36-45.
23. Johne R, Wittig W, Fernandez-de-Luco D, Hofle U, Muller H (2006) Characterization of two novel polyomaviruses of birds by using multiply primed rolling-circle amplification of their genomes. J Virol 80: 3523-3531.
